# Interdependence of PRC1 and PRC2 for recruitment to Polycomb Response Elements

**DOI:** 10.1093/nar/gkw701

**Published:** 2016-08-23

**Authors:** Tatyana G. Kahn, Eshagh Dorafshan, Dorothea Schultheis, Aman Zare, Per Stenberg, Ingolf Reim, Vincenzo Pirrotta, Yuri B. Schwartz

**Affiliations:** 1 Department of Molecular Biology, Umeå University, Umeå, 901 87, Sweden; 2 Friedrich-Alexander University of Erlangen-Nürnberg, Department of Biology, Division of Developmental Biology, Erlangen, D-91058, Germany; 3 Division of CBRN Defense and Security, Swedish Defense Research Agency, FOI, Umeå, 906 21, Sweden; 4 Department of Molecular Biology and Biochemistry, Rutgers University, Piscataway, NJ 08854, USA

## Abstract

Polycomb Group (PcG) proteins are epigenetic repressors essential for control of development and cell differentiation. They form multiple complexes of which PRC1 and PRC2 are evolutionary conserved and obligatory for repression. The targeting of PRC1 and PRC2 is poorly understood and was proposed to be hierarchical and involve tri-methylation of histone H3 (H3K27me3) and/or monoubiquitylation of histone H2A (H2AK118ub). Here, we present a strict test of this hypothesis using the *Drosophila* model. We discover that neither H3K27me3 nor H2AK118ub is required for targeting PRC complexes to Polycomb Response Elements (PREs). We find that PRC1 can bind PREs in the absence of PRC2 but at many PREs PRC2 requires PRC1 to be targeted. We show that one role of H3K27me3 is to allow PcG complexes anchored at PREs to interact with surrounding chromatin. In contrast, the bulk of H2AK118ub is unrelated to PcG repression. These findings radically change our view of how PcG repression is targeted and suggest that PRC1 and PRC2 can communicate independently of histone modifications.

## INTRODUCTION

Polycomb Group (PcG) proteins are evolutionarily conserved transcriptional regulators essential for development of all complex plants and animals. PcG proteins act in concert to epigenetically repress multiple master regulatory genes thereby enforcing cell-type specific gene expression programs (1). PcG proteins form complexes of two kinds: PRC1 and PRC2. PRC2 complexes methylate Lysine 27 of histone H3 (2–5) and extensive tri-methylation of H3K27 (H3K27me3) at PcG target genes is essential for repression (6). There are two flavors of PRC2 complexes. Both contain an invariant core of five proteins E(z), Esc, Su(z)12, Caf1 and Jing (here listed by their names in *Drosophila melanogaster*) but differ in the presence of alternative subunits Jarid2 or Pcl (7). In *Drosophila melanogaster*, the PRC2–Pcl complexes act at PcG target genes (8) while the function of PRC2–Jarid2 complexes is not yet clear. In addition to their role at PcG target genes, PRC2 complexes are also responsible for pervasive di-methylation and scattered low-level tri-methylation of H3K27 throughout the entire transcriptionally inactive genome (9,10). This genome-wide H3K27 methylation arises from untargeted ‘hit-and-run’ action whose mechanistic details are not well understood.

The PRC1 class is less well defined and its systematics is not fully settled (7,11). Here, we reserve the name PRC1 for complexes that, in *Drosophila*, consist of Pc, Ph and Scm proteins along with a heterodimer between RING1 (the product of the *Sce* gene) and one of the two closely related PCGF proteins: Psc or Su(z)2 (12–14). Mutations in genes encoding subunits of PRC1 lead to embryonic lethality and mis-expression of *HOX* genes, indicating that this complex is essential for PcG repression. In addition to PRC1, the RING1-Psc dimers are incorporated into a different complex called dRAF. The dRAF complex lacks Pc, Ph and Scm subunits and instead contains histone demethylase Kdm2, RAF2 and Ulp1 proteins (15). Both PRC1 and dRAF can monoubiquitylate Lysine 118 of histone H2A (H2AK118ub, the analog of mammalian H2AK119ub) via the RING-PCGF catalytic core (15,16). dRAF was reported to produce the bulk of *Drosophila* H2AK118ub and mutations in the *Kdm2* gene were said to enhance the homeotic phenotypes of heterozygous *Pc* mutants (15). However, a recent study suggests that Kdm2 is not essential for fly viability (17) questioning the importance of dRAF for PcG repression. Finally, *Drosophila* RING1 can potentially form a dimer with a third *Drosophila* PCGF protein called L(3)73Ah. Although this interaction has yet to be demonstrated biochemically, L(3)73Ah plays an important role in global H2A118 ubiquitylation (10).

In mammals, the repertoire of PCGF proteins is expanded to six and there are two closely related RING1 proteins: RING1 and RING2 (7). Heterodimers between RING2 (or RING1) and MEL18 (a.k.a. PCGF2) or BMI1 (a.k.a. PCGF4) are incorporated in the complexes analogous to *Drosophila* PRC1 (sometimes referred to as canonical mammalian PRC1). Like *Drosophila* PRC1, these complexes also include one of the five variant chromodomain proteins (CBX2, CBX4, CBX6, CBX7 or CBX8), one of the three Polyhomeotic-like proteins (PHC1, PHC2 and PHC3) and SCMH1 (or related SCML2) protein (11). Mutations in genes encoding subunits of the canonical mouse PRC1 lead to embryonic lethality and mis-expression of *HOX* genes, indicating that these complexes are essential for PcG repression (18–21). RING2 or RING1 dimerized with one of the four other PCGF proteins (PCGF1, PCGF3, PCGF5 or PCGF6) form complexes that contain RYBP (or closely related YAF2 protein) instead of CBX and PHC subunits. One of these complexes, which incorporates the RING2–PCGF1 dimer and the KDM2B subunit, clearly contributes to PcG repression (22–24). The role of the other complexes is not yet clear and some of them have functions unrelated to PcG repression (25).

*Drosophila* PRC1 and PRC2 are targeted to specific genes via Polycomb Response Elements (PREs). These ∼1 kb long DNA elements correspond to genomic high-affinity binding sites for PRC1 and PRC2. When tested in transgenic experiments, PREs are necessary and sufficient to recruit these complexes and their repressive activities to reporter genes. PREs contain collections of recognition motifs for multiple sequence-specific DNA binding proteins. These DNA binding proteins do not stably associate with PRC1 or PRC2 complexes but act as adapters that combine individually weak interactions to tether PRC complexes to PREs (26). A targeting based on combinatorial interactions provides a way to attenuate the repression of target genes in cells where those must remain active. However, it raises the question of how the recruitment of the PRC complexes is coordinated.

PRC1, but not dRAF or any of the mammalian RING-PCGF-RYBP complexes, can specifically recognize H3K27me3 produced by PRC2 via the chromodomain of its Pc subunit (27). PRC2 can specifically recognize H2AK119ub, produced by PRC1 (28). This led to hypotheses that these histone modifications are important to coordinate the recruitment (22,29). During replication, nucleosomes are randomly partitioned between the daughter chromosomes so it is easy to see how the modified histones could serve as epigenetic memory by facilitating the binding of PRC complexes to genes that have been repressed in a previous cell cycle. Yet, the experimental evidence supporting the model is weak. It was noted that PREs, the principal binding sites of *Drosophila* PRC complexes, are often depleted of nucleosomes and hence of H3K27me3 (30–32). More recently, the studies from the Klose and Brockdorff labs showed that in mouse embryonic stem cells, ubiquitylation of H2AK119 by a RING2-PCGF1-KDM2B complex is necessary and sufficient to recruit PRC2 and H3K27me3 to multiple target genes (22,33). This supports the role of H2AK119ub as a key component of the recruitment hierarchy. However, similar experiments in human HEK293T cells did not detect the recruitment of PRC2 by H2AK119ub (24) raising the question of whether this targeting mechanism operates in all cell types. The importance of H2AK119ub for PcG repression is further questioned by the reports that catalytically inactive RING2-complexes are still able to repress mouse *HOX* genes (34) and support early embryonic development (35) and that in H2AK118ub-deficient flies PcG repression is not impaired (36). Paradoxically, in *Drosophila*, the H2AK119 deubiquitylating complex PR-DUB is also required for PcG repression of the *HOX* genes (37). Therefore, the role of H3K27me3 and H2AK118ub and the interdependence between PRC1 and PRC2 in coordinated recruitment remain key unanswered questions.

Here, we use the *Drosophila* model to address this issue. To our advantage, the repertoire of the *Drosophila* RING-PCGF complexes is smaller than in mammals, most of the PcG proteins are non-redundant and the DNA elements involved in the recruitment are well defined. Using custom genetically engineered cultured cell lines we discover that both H3K27me3 and H2AK118ub are dispensable for targeting PcG complexes to PREs. We find that most PREs require the presence of PRC1 or dRAF to recruit PRC2, although at some PREs, PRC2 and PRC1 are recruited independently. In contrast, PRC1 does not require PRC2 to bind PREs, although both are required for effective repression at most sites. We provide the evidence that H3K27me3 helps PcG complexes anchored at PREs to interact with surrounding chromatin. We also find that the bulk of histone H2AK118ub is produced by RING1-L(3)73Ah complexes at sites other than PcG target genes and that the levels of this H2AK118ub are kept in check by the deubiquitylating activity of PR-DUB. Our results indicate that there are alternative ways to target PcG repression to genes and suggest that PRC1 and PRC2 communicate independently of histone modifications.

## MATERIALS AND METHODS

### Derivation of cultured cell lines

The *Su(z)2-1.b8* and *Su(z)12^4^* fly lines used to derive corresponding cell lines have been described by (38,39). Sequencing of chromatin Input samples allowed us to determine the precise positions of *Su(z)2-1.b8* deletion breakpoints. According to this analysis the deletion removes the part of Chromosome 2R between nucleotides 8 828 024–9 009 615 in Dm3 (2006) genome release coordinates. The cultured cells were derived following the procedure of Simcox *et al*. (40) with some modifications. The chromosome carrying corresponding mutation was recombined with a transgene encoding RAS^V12^ driven by *UAS* promoter or with a transgene expressing high level of GAL4 from constitutively active *Act5C* promoter. The recombination events were confirmed by crossing to fly stocks with unrelated *Psc^h27^* or *Su(z)12^3^* mutant alleles and sequencing. The heterozygous fly stocks bearing recombinant chromosomes were further crossed to each other to yield embryos that carry homozygous mutation and a combination of *UAS-RasV12* and *Act5C-GAL4* transgenes. A total of 0.1 g of overnight embryos from each cross were collected at 17°C on apple juice plates supplemented with heat-inactivated yeast paste, rinsed in a sieve, dechorionated in 14% bleach for 3–5 min, washed extensively with sterile TXN (0.02% Triton X-100; 0.7% NaCl) and transferred to a 15 ml sterile conical tube. After rinsing with 1x PBS, the embryos were homogenized with 3 gentle strokes in 3 ml of Schneider's cell culture medium (Lonza), supplemented with 10% heat-inactivated fetal bovine serum (FBS) (Sigma) and 1/100 dilution of streptomycin–penicillin solution (Invitrogen) under sterile conditions. After large cell clumps and unbroken embryos had settled down the supernatant was transferred to a 15 ml conical tube. Remaining embryos and tissue clumps were homogenized in a second aliquot of same culture medium with slightly firmer strokes and added to homogenate. Cells were pelleted by centrifugation, rinsed with three changes of culture medium and plated in three 25cm^2^ T-flasks at 25°C. Cultured medium was changed once a week and confluent cultures were passaged after trypsin treatment. Parent cultures were maintained for as long as possible (by supplying fresh cultured medium to the cells that remained attached to the surface of the flask following trypsin treatment) and used to establish multiple first passage cultures until several cell lines showed successful continued growth. To inhibit transcriptional elongation, Psc/Su(z)2 mutant cells were treated with 100 μM 5,6-dichloro-1-beta-D-ribofuranosylbenzimidazole riboside (Sigma) in 1% DMSO in Drosophila Schneider's complete medium for 12 h at +25°C. After 12 h cells were collected for Western blot, RT-PCR and Chromatin immunoprecipitation (ChIP) analyses. In parallel Psc/Su(z)2 minus cells were treated with 1% DMSO as a control. The growth and ds-RNA treatment of S2-DRSC cells were done as described (41) and the primers used for dsRNA synthesis are listed in the Supplementary Table S1.

### Antibodies and Western blot analysis

The antibodies used in this study are listed in the Supplementary Table S2. Rabbit polyclonal antisera against Ash1 protein was raised against the peptide containing amino acids 1756–1855 fused to GST. The antibodies were further affinity purified as described by Poux *et al*. (42) and are available for purchase through Agrisera, cat. number: AS14 2816. Total nuclear protein was isolated by first lysing cells in hypotonic buffer containing 10% sucrose, 10 mM Tris pH8.0, 10 mM NaCl, 3 mM MgCl_2_, 2 mM DTT and 0.2% Triton X100, followed by 10 min extraction of the nuclear pellet with Sample Buffer (12 mM Tris-HCl pH6.8, 5% glycerol, 0.4% SDS, 2.9 mM 2-mercaptoethanol, 0.02% bromphenol blue) at 100°C. To isolate histones, the nuclear pellet from above was partially depleted of non-histone proteins by sequential extraction with buffered 350 mM and 600 mM NaCl solutions. The histones were taken in 0.2 M sulfuric acid, TCA precipitated and dissolved in 0.1 N NaOH. Serial dilutions of protein samples were loaded on SDS polyacrylamide gel, separated by electrophoresis, transferred to a PVDF membrane and detected by incubation with primary antibodies for 1 h at room temperature followed by 30 min incubation with secondary antibodies conjugated with alkaline phosphatase.

### Expression analysis

Total RNA from 5 × 10^6^ cells was isolated using Trizol (Invitrogen) and 2 μg were used for random primed synthesis of cDNA with RevertAid First Strand cDNA Synthesis Kit (Thermo Scientific). The control reaction, omitting reverse transcriptase, was always run in parallel. The cDNA was purified with DNA Clean and Concentrator Kit (Zymo Research), eluted with 100 μl of elution buffer and analyzed by qPCR. The sequences of primers are given in Supplementary Table S1. Serial dilutions of genomic DNA were used to make the standard curve. The amount of cDNA for a gene of interest in a given preparation was expressed as a fraction of *RpL32* cDNA.

### Chromatin immunoprecipitation and genome-wide analyses

ChIP and quantitative polymerase chain reaction (qPCR) analysis were done as described (32). To prepare cross-linked chromatin from transgenic fly strains, 500 mg of 3rd instar larvae were collected, grinded in liquid nitrogen and cross-linked for 20 min in 20 ml of 1.8% formaldehyde, 50 mM Hepes pH8.0, 1 mM ethylenediaminetetraacetic acid (EDTA) pH8.0, 0.5 mM EGTA pH8.0, 100 mM NaCl at +25°C. Reaction was stopped by adding 2.2 ml of 1.25 M Glycine pH7.0 and incubating at +4°C for 5 min. Cross-linked material was washed in 10 mM Hepes pH7.6, 10 mM EDTA pH8.0, 0.5 mM EGTA pH8.0, 0.25% Triton X-100 for 10 min at +4°C followed by washing in 10 mM Hepes pH7.6, 1 mM EDTA pH8.0, 0.5 mM EGTA pH8.0, 100 mM NaCl, 0.01% Triton-X100 for 10 min at +4°C. Chromatin was sonicated in 4 ml of 10 mM Tris-HCl pH8.0, 1 mM EDTA pH8.0, 0.1% SDS, adjusted to 5 ml of RIPA as described (32), separated from insoluble material by centrifugation at 12 000 *xg* for 5 min at +4°C and 0.5 ml aliquots were used for one ChIP. The primers used for qPCR are listed in Supplementary Table S1.

The libraries for sequencing ChIP products and corresponding chromatin Input DNA were prepared using NEBNext ChIP-Seq Library Prep Master Mix Set and Multiplex Oligos for Illumina (New England BioLabs) following the manufacturer instructions. Two to four libraries were pooled and sequenced with MiSeq instrument (Illumina) using MiSeq Reagent Kit and corresponding run protocol. This yielded 3.2–15 million reads per library of which >80% were unique. The reads were aligned to the Dm3 2006 *Drosophila* reference genome with bowtie2 (43) using default parameters. The reads were tested for strand correlation, extended accordingly and read density profiles generated using Pyicos (44).

To compare the E(z) or Psc ChIP-seq signals at PREs in different cell lines, the read density profiles were normalized to the total sequencing depth and the mean read density for 6 consecutive positions with the highest read counts within 1 kb PRE fragments were computed using RegionMeanSameSort6 script (Supplementary File 1). These values showed >90% correlation between the replicate experiments and were further averaged to calculate relative differences. The list of PREs used for these analyses (Supplementary Table S3) was derived as follows. PREs computationally defined in (45) were filtered to remove regions overlapped by *Su(z)2-1.b8* deletion as well as regions with weak E(z) ChIP-seq signals in Ras3 cells (lower 30% of signal dynamic range). The boxplots and histograms were produced using generic R (www.R-project.org) functions ‘boxplot’, ‘hist’ and ‘density’. The Wilcoxon rank sum tests were performed using generic R function ‘wilcox.test’. Violin plots were produced using a R ‘vioplot’ package from Daniel Adler.

Cumulative Pc distributions around isolated Psc peaks in Su(z)12 deficient and control Ras3 cells were calculated as follows. Psc peaks were defined as centers of six consecutive coordinates with the highest read counts within significantly immunoprecipitated regions. Significantly immunoprecipitated regions were defined as clusters of coordinates that are no more than 500 bp apart and whose values exceed three Standard Deviations of genomic mean. The 10 kb regions centered on Psc peaks were divided in 100 bp bins for which the average read counts were calculated for each of the replicate ChIP and Input sequencing experiments. The resulting values were averaged between corresponding replicate experiments and used to fit the smooth curve with loess algorithm and span parameter of 0.1. The difference between cumulative distributions was calculated based on subtraction of values for 50 points evenly distributed on fitted curves. These values were used to fit a smooth curve with loess algorithm and span parameter of 0.2. Average read counts outside PcG target genes were calculated by taking the mean of average read counts within 999 randomly chosen 100 bp bins that are not overlapped by regions bound by Psc in Ras3 cells. Fitting and plotting of smooth curves was done with ‘ggplot’, ‘theme_classic’, ‘stat_smooth’ and ‘geom_hline’ functions of the ‘ggplot2’ R package by Hadley Wickham.

### Transgenic work

The constructs to test the functional properties of *Doc1* and *Doc3* PREs were made as follows. DNA fragments were generated via PCR using BACR10P09-derived larger fragments as a template and cloned into the expression analysis P-element vectors described by Barolo *et al*. (46). Doc1 fragment was amplified using primers 5′-ATTGCATGTT TTCAATTTGC GTTGATG-3′ and 5′-GCTCCGCTCG AGTGCTAACT CGCCGT-3′ and cloned from a pCR-TOPO intermediate into the *Eco*RI site of pH-Pelican. Doc3 fragment was generated using restriction site-tagged primers 5′-GACGTCTAGA GAGTTAACGA AGATTTTCCA ATCTGTTT-3′ and 5′-TAATGAATTC CGTGGGCAAT CGACGTCT-3′ and cloned between the *Xba*I and *Eco*RI sites of pPelican. The transgenic flies were obtained by injecting the DNA into preblastoderm embryos of *w^−^* or *y^−^, w^−^* genotype and selecting for the progeny with colored eyes. Two transgenic lines *Doc F3s1-1b* and *DocF3s1-4* here referred to as *Doc 1 line 1* and *Doc 1 line 2* and three transgenic lines *DocF11s1-3, DocF11s1-9, DocF11s1-10* here referred to as *Doc 3 line 1, Doc 3 line 2* and *Doc 3 line 3* were used for further experiments. To introduce *su(Hw)* mutant background the males containing transgenic insertions on the second chromosome were crossed to *y^1u1^,sc^D1^,w^1118^; +/+; su(Hw)^v^/TM6, su(Hw)^f^* females and the progeny screened for *y^1u1^,sc^D1^,w^1118^/Y; Transgene[w^+^]/+; +/TM6, su(Hw)^f^* males that lack bristles on scutellum and colored eyes. Such males were further crossed to *y^1u1^,sc^D1^,w^1118^; +/+; su(Hw)^v^/TM6, su(Hw)^f^* to yield heterozygous *y^1u1^,sc^D1^,w^1118^; Transgene[w^+^]/+; su(Hw)^v^/TM6, su(Hw)^f^* flies. Males and females of the above genotype were interbred to yield the flies with two copies of a transgene on *su(Hw)* mutant background.

To generate the pWattB-2FRT-gypsy-PRE constructs the *gypsy* insulator fragment was excised from 2xR-suHw plasmid (47) and cloned between the two FRT sequences. The FRT-gypsy cassette was further cloned into Eco47III site of pWattB plasmid (generous gift of Dr Mikhail Savitsky) resulting in pWattB-2FRT-gypsy construct. *bxd*-PRE fragment was PCR amplified from genomic DNA using BXD-PRE-FRT-1 (5′-ATCCACTAGTTCTAGGAAGTTCGCCTGTTGCCTTG-3′) and attB-BXD-PRE-1 (5′- TGGCGGCCGCTCTAGGTTTTATGCTGCCCGCTTGT-3′) primers and cloned into XbaI site of the pWattB-2FRT-gypsy construct. *HGTX* PRE, *knrl* PRE and the intergenic control fragments were amplified with HGTX-PRE-FRT-1 (5′-ATCCACTAGTTCTAGAGAAAGGCCCAAGGCAACTT-3′) and attB-HGTX-PRE-1 (5′- TGGCGGCCGCTCTAGTTGGGTAAGCTCAGACAGGC-3′), knrl-PRE-FRT-1 (5′- ATCCACTAGTTCTAGATTGCACAGTTTATTTCTCGGTT-3′) and attB-knrl-PRE-1 (5′-TGGCGGCCGCTCTAGTGACCGATACTACCAACGGC-3′), 3Lnc-FRT-1 (5′-ATCCACTAGTTCTAGGACTCTGCTCGCCTCGTATC-3′) and attB-3Lnc-1 (5′-TGGCGGCCGCTCTAGTGCTATCGTGTCGCTCCATC-3′) primers respectively and cloned into pWattB-2FRT-gypsy as described for *bxd*-PRE. To obtain transgenic flies the DNA of pWattB-2FRT-gypsy-PRE constructs was injected into preblastoderm embryos of y^1^, M[vas-int.Dm]ZH-2A w*; M[3xP3-RFP.attP]ZH-51C genotype (48). The emerging adults were crossed to *y^1^, w^1118^* flies and the transgenic progeny identified by eye color. To ‘excise’ the *gypsy* insulator element from the FRT-cassette, the transgenic flies were crossed to *y^1^,w^−^, P[ry[+t7.2] = 70FLP]3F / Dp(1:Y)y^+^; Sco/SM6a, CyO* and the expression of FLP-recombinase induced by two 2-h heat-shock treatments (37°C) during the 3rd and the 4th day after egg deposition.

To document the expression of the *white* gene 5-day-old flies were deeply anesthetized with diethyl ether and their eyes photographed at 7x magnification using SMZ 1500 microscope and DS-Fi1 CCD camera (Nikon). For each picture seven to ten Z-stacks were collected and combined using NIS-Elements BR software (Nikon). At least three different flies of each genotype were photographed and a representative image used for illustration.

## RESULTS

**Figure 1.**
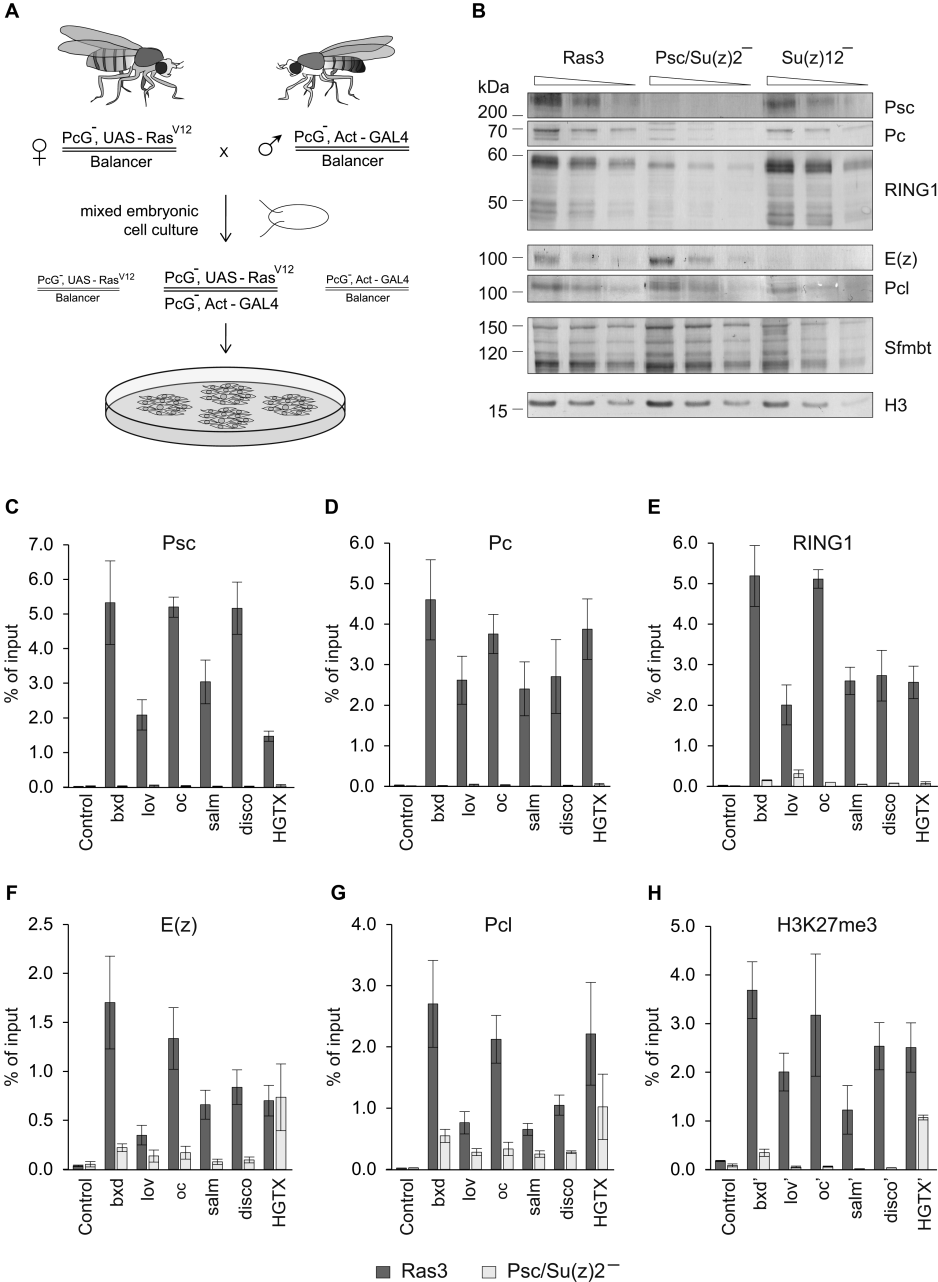
Effects of PRC1 ablation on H3K27me3 and PRC2 binding at PREs. (**A**) Derivation of cultured cell lines lacking PcG proteins. Chromosomes with null mutations in genes encoding PcG proteins were recombined with a transgene encoding RAS^V12^ driven by *UAS* promoter or with a transgene expressing transcriptional activator GAL4 from strong *Act5C* promoter. Primary cell culture from embryos collected after crossing the recombinant fly stocks contains a mixture of cells with different genotypes. However, only cells homozygous for PcG mutation contain the combination of RAS^V12^ and GAL4 transgenes that lets them to proliferate in culture. (**B**) Two-fold dilutions of total nuclear protein from control (Ras3) and mutant cell lines were analyzed by Western-blot with antibodies against key components of PRC1, PRC2 and PhoRC complexes (indicated to the right). Positions of relevant molecular weight markers (in kDa) are shown to the left of each panel. Note that the ablation of Psc/Su(z)2 strongly reduces the overall levels of other PRC1 components but does not affect the levels of PRC2 and PhoRC. Conversely, the ablation of Su(z)12, affects the components of PRC2 but not of PRC1 or PhoRC. (**C–H**) Chromatin Immunoprecipitation coupled to quantitative PCR (ChIP-qPCR) analyses of PcG proteins and H3K27me3 at a set of PREs in control (Ras3) and Psc/Su(z)2 minus cells. Here and below the mean of two independent experiments and the scatter (error bars) are shown. The ‘Control’ amplicon corresponds to an intergenic region on Chromosome 3L that does not bind PcG proteins. Since PREs are often depleted of nucleosomes the H3K27me3 levels were assayed at amplicons (marked with apostrophe) ∼2 kb to the side of PRE cores. While at most PREs loss of PRC1 leads to the loss of PRC2 and H3K27me3, *HGTX* PRE stands out by retaining significant amount of both.

To understand the interdependence between PRC1 and PRC2 and the role of H3K27me3 and H2AK118ub in the targeting of PcG complexes we generated *Drosophila* cultured cell lines that lack critical subunits of PRC1 or PRC2 (Figure 1A). To this end we used *Drosophila* fly stocks expressing constitutively active RAS^V12^ protein (40) and derived two independent cultured cell lines homozygous for *Su(z)2-1.b8* deficiency and four independent cultured cell lines homozygous for the *Su(z)12^4^* mutation. The *Su(z)2-1.b8* mutant cells carry 180.6 kb deletion that removes the entire transcription units of the closely related *Psc* and *Su(z)2* genes and produce no Psc and Su(z)2 proteins (39). The *Su(z)12^4^* mutation is a single nucleotide substitution that results in the premature translation termination and a short non-functional protein product (38). Although both Psc/Su(z)2 and Su(z)12 proteins are essential for PcG repression and flies homozygous for corresponding loss-of-function mutations die during embryogenesis (38,39), the mutant cells are viable and proliferate in culture. Therefore we obtained an experimental system that avoided the redundancy of mammalian PcG group family, provided large quantities of material for biochemical experiments and allowed us to assay the effect of the loss of individual PcG complexes without simultaneous activation of target genes by developmental enhancer factors. The latter is critical to uncouple the direct dependency between PRC complexes from changes triggered by transcription and Trithorax Group (TrxG) proteins (49–53).

### PRC2 binding to the majority of *Drosophila* Polycomb Response Elements depends on PRC1

As expected, in the cells derived from *Su(z)2-1.b8* mutant embryos Psc protein is not detectable (Figure 1B). Here and below, we present experiments with cells of the *Psc4-1* line, hereafter referred as Psc/Su(z)2 minus cells. However, we obtained essentially the same results with the independently derived *Psc3-1* cell line carrying the same mutation, indicating that effects are not specific for a particular cell isolate. The loss of Psc/Su(z)2 proteins is accompanied by a concomitant 4-fold reduction in the overall level of RING1 protein (Figure 1B). Since in Psc/Su(z)2 minus cells the transcription of the *Sce* gene encoding RING1 does not decrease compared to that in the control cell line (Supplementary Figure S1), this suggests that the bulk of the RING1 protein is in a complex with Psc or Su(z)2 and that in their absence RING1 is unstable and degraded. The overall level of Pc protein, but not its transcript, is also strongly reduced (Figure 1B, Supplementary Figure S1). This indicates that when Psc and Su(z)2 are absent the canonical PRC1 complex disintegrates. In contrast, the overall levels of PRC2 subunits E(z) and Pcl or the Sfmbt subunit of the auxiliary PhoRC complex (45,54), are not affected by the loss of Psc and Su(z)2 (Figure 1B), suggesting that the integrity of PRC2 and PhoRC is not dependent on PRC1 or dRAF.

We next asked whether the binding of PRC2 to PREs is affected by the loss of PRC1 or dRAF. As a first step, we assayed the binding of PRC1 and PRC2 in Psc/Su(z)2 and control RAS^V12^ transformed but otherwise wild-type cells (Ras3 cell line) at 6 PREs from our computationally defined high-confidence list (Supplementary Table S3, (45)). These PREs were selected to cover a broad range of distances (25–0.5 kb) to Transcription Start Sites (TSS) of likely target genes and included the well-characterized *bxd*-PRE of the homeotic *Ultrabithorax* (*Ubx*) gene, as well as the putative PREs of *jim lovell* (*lov*), *ocelliless* (*oc*), *spalt major* (*salm*), *disconnected* (*disco*) and *HGTX* developmental genes (Supplementary Figure S2).

As anticipated, in the Psc/Su(z)2 mutant cells Chromatin Immunoprecipitation coupled to quantitative PCR (ChIP-qPCR) detects no presence of Psc or Pc proteins at PREs and very little, if any, RING1 (Figure 1C–E). Remarkably, in the absence of PRC1 the ChIP signals for PRC2 components E(z) and Pcl are also dramatically reduced (∼10-fold) at five out of six tested PREs (Figure 1F–G). With the notable exception of *HGTX* PRE, whose precipitation with antibodies against PRC2 subunits is largely unaffected by the ablation of PRC1 (Figure 1F–G), the loss of PRC2 from PREs is accompanied by dramatic reduction of H3K27me3 in their vicinity (Figure 1H). These observations indicate that in many cases the binding of PRC2 to PREs depends on the presence of PRC1 or dRAF and suggest that PREs differ in how they coordinate the recruitment of PRC1 and PRC2.

### Genome-wide picture of PRC2:PRC1 dependence

**Figure 2.**
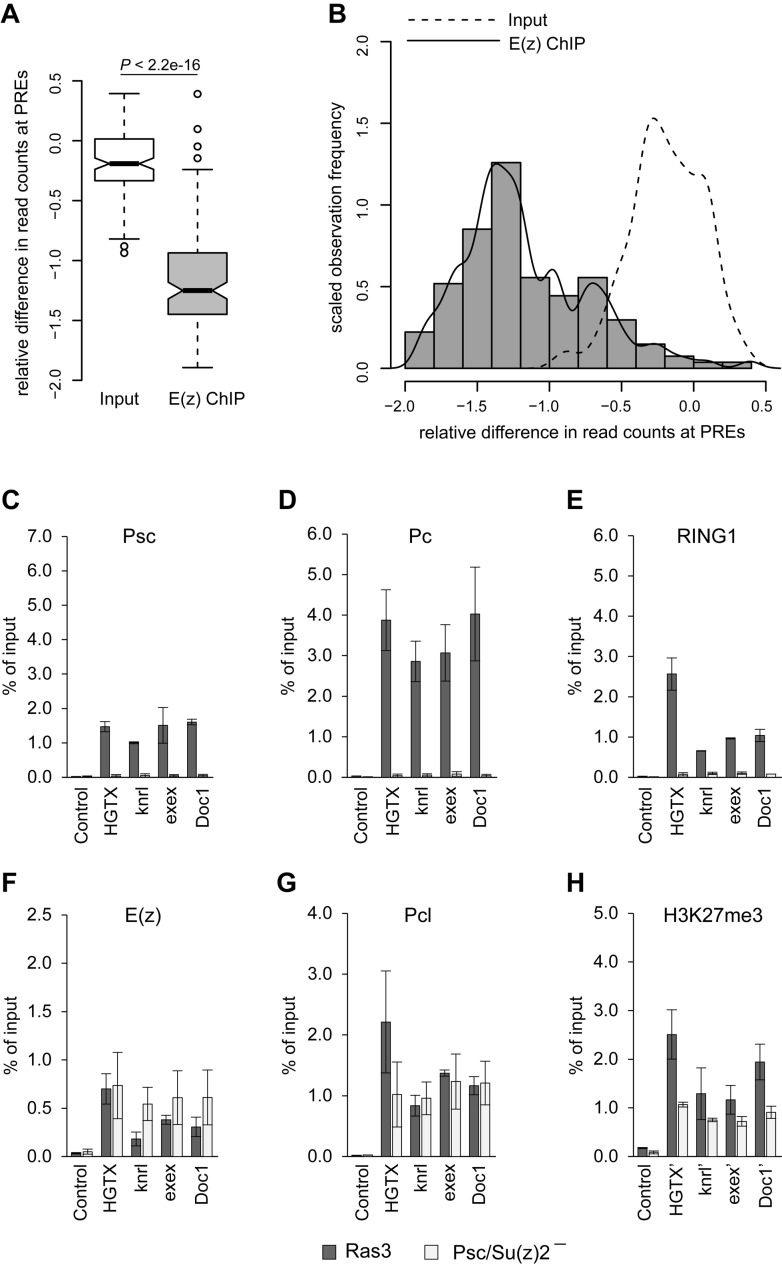
Overview of PRC2:PRC1 dependence at PREs. (**A**) The box-plots compare the relative difference in the average number of sequence read counts at PREs (n = 135) measured by E(z) ChIP-seq experiments with Psc/Su(z)2 minus and Ras3 control cells (grey boxplot, ‘E(z) ChIP’) to the relative difference of in the average number of read counts after sequencing corresponding chromatin input samples (white boxplot, ‘Input’). The boxplots indicate the median and span the inter-quartile range. The whiskers indicate the lowest and the highest values excluding outliers, which are defined as values outside of 1.5 time inter-quartile range. Notches correspond to the 95% confidence intervals of the median values. At most PREs the loss of ChIP-seq signal in Psc/Su(z)2 minus cells is greater than variability between Input sequencing experiments and the overall difference is statistically significant (Wilcoxon rank sum test). (**B**) The histogram and the solid density fitting curve illustrates the distribution of relative differences in the number of E(z) ChIP-seq read counts (RD) at different PREs between Psc/Su(z)2 minus and Ras3 control cells. The long right tail of the distribution overlaps with the distribution of relative differences of the read counts in the control Input sequencing experiments (dashed curve). All PREs with RD < −1.0 show clear PRC2:PRC1 dependence. The PREs with RD > −0.5 are likely to be PRC2:PRC1-independent. The observation frequencies (y-axis) are scaled such that the total area underneath the histograms equals one. (**C–H**) ChIP-qPCR analyses of PcG proteins and H3K27me3 at a set of PRC2:PRC1 independent PREs in control (Ras3) and Psc/Su(z)2 minus cells.

What fraction of PREs can recruit PRC2 independently of PRC1? To address this question we compared the binding of the E(z) subunit of PRC2 at PREs in Psc/Su(z)2 deficient and control RAS^V12^ transformed cells using ChIP coupled to next generation sequencing of the precipitated DNA (ChIP-seq). We did two independent ChIP-seq experiments for each genetic background and had the DNA from corresponding chromatin input materials sequenced to compensate for potential sample processing biases. The comparison of ChIP-seq signals at computationally defined PREs (45) indicates that the replicate experiments are highly concordant (Pearson's product-moment correlation >0.90; *P*-value < 2.2e-16). The majority of PREs show significantly fewer sequencing reads in ChIP samples from Psc/Su(z)2 deficient cells compared to those from control cells (Figure 2A, Supplementary Table S3) but some show little change (Figure 2B). The ChIP-qPCR analysis of three representative PREs from the *Doc1* (relative difference in read counts RD = −0.15), *exex* (RD = −0.24) and *knrl* (RD = −0.31) genes, which show little change in Psc/Su(z)2 deficient cells, validates the ability of ChIP-seq to distinguish PREs based on E(z) loss upon PRC1 ablation (Figure 2C–H). The overall comparison of the E(z) ChIP-seq signal loss at different PREs to differences in read counts between corresponding chromatin input samples suggests that less than one-third of all PREs can bind the full extent of PRC2 when PRC1 is absent (Figure 2B). We note that although PREs from the middle of the histogram on Figure 2B show significant loss of the E(z) ChIP-seq signals, these signals in the PRC1-deficient cells remain above the background levels. This suggests that PRC1-dependent and -independent pathways are not mutually exclusive and can combine their inputs to recruit PRC2 to some PREs.

Multiple lines of evidence indicate that the loss of PRC2 from PREs in PRC1-deficient cells is not due to indirect effects of increased transcriptional activity or counteracting action of TrxG proteins. First, transcription through PREs in the mutant cells is exceedingly low and remains the same as in the control cells regardless of their PRC2:PRC1 dependence status (Supplementary Figure S3A). Second, although in Psc/Su(z)2 minus cells the transcription of corresponding target genes generally increases compared to that at the control cells (Supplementary Figure S3B, (10)), the fold and the amplitude of the change varies greatly among the loci (Supplementary Figure S3B) and is not correlated with the degree of PRC2 loss. Third, in most cases the loss of PRC1 is not accompanied by an increase in Ash1 binding at the PREs (Supplementary Figure S3C), the hallmark of de-repressed chromatin state associated with the action of TrxG proteins (31,41,55). Fourth, the ablation of PRC1 specifically affects the binding of PRC2 to PREs but not its general activity. This follows from the observations that in Psc/Su(z)2 deficient cells the overall levels of H3K27me3 and H3K27me2 are not significantly altered (Supplementary Figure S4A) and the H3K27me3 around PRC2:PRC1-dependent PREs is replaced by H3K27me2 (Supplementary Figure S4B). The latter indicates that in the Psc/Su(z)2 mutant cells there is no generic activity that makes the chromatin around PRC2:PRC1-dependent PREs ‘repulsive’ to PRC2. It also argues that in these cells there is no transcription around PREs as H3K27me2 is generally excluded from transcriptionally active regions (10).

Finally, to test the possibility that the loss of PRC2 from PRC2:PRC1-dependent PREs is due to transcription of an RNA that is hard to detect, we assayed the binding of E(z) to PREs after inhibiting the transcription with 5,6-dichloro-1-beta-D-ribofuranosylbenzimidazole riboside (DRB). DRB inhibits transcript elongation by suppressing Serine 2 phosphorylation (Ser2-P) of RNA Polymerase II (RNAPII) and Riising *et al*. (56) recently showed that inhibiting transcriptional elongation in mouse embryonic stem cells induces the binding of PRC2 to CpG-rich DNA sequences. Treatment of the Psc/Su(z)2 deficient cells with 100 μM DRB resulted in ∼10-fold reduction of RNAPII Ser2-P without reducing the overall RNAPII level (Supplementary Figure S5A and B). RT-qPCR analysis of unspliced RNAs from house-keeping *Taf4* gene and derepressed PcG target *oc* and *srp* genes showed that transcription was robustly inhibited (Supplementary Figure S5C). In contrast, we detected no increase in E(z) ChIP signals at both PRC2:PRC1-dependent and independent PREs in DRB-treated cells compared to vehicle-treated control (Supplementary Figure S5D). Taken together our observations argue that, in most cases, the physical presence of PRC1 (or dRAF) at PREs is required for PRC2 to bind efficiently.

### PRC2:PRC1-independent PREs can autonomously recruit PcG complexes and repress a reporter gene

**Figure 3.**
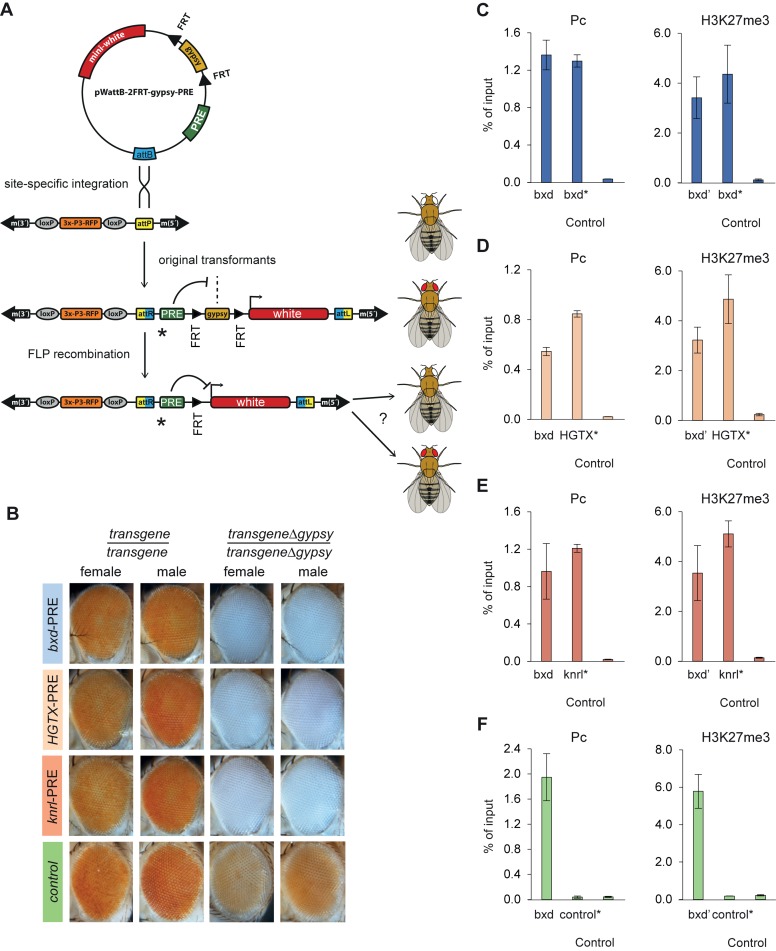
Comparison of PRC2:PRC1 dependent and independent PREs integrated in the same chromatin environment. (**A**) Schematic of the experimental setup. Tested PREs or a negative control fragment (green rectangle) were cloned into the pWattB-2FRT-gypsy vector that contained attB site (blue) for ϕC31-mediated recombination with attP site (yellow) of the ZH-51C landing site. In addition the vector contained the *white* reporter gene (red rectangle) separated from PREs by an FRT cassette (black triangles) with the *gypsy* insulator element. The flies with ZH-51C landing site have white eyes marked by RFP. Upon recombination the eye color of the flies turns orange because potential repression of the *white* gene by PREs is prevented by *gypsy* insulator. If a PRE under study is able to repress the *white* gene the FLP-mediated excision of *gypsy* insulator will lead to reduced eye coloration, otherwise the eyes will remain orange. Asterisks indicate the position of PCR amplicons spanning the junctions between transgenic PREs and adjacent vector DNA used to analyze the immunoprecipitation of transgenic PREs. (**B**) Expression of *white* reporter gene in transgenic lines. The eye colors of male and female flies homozygous for the original transgenic insertions are compared to that of the corresponding homozygous transgenic flies from which the *gypsy* insulator cassette was excised by FLP recombination. (**C–F**) ChIP-qPCR analyses of Pc and H3K27me3 within transgenic (C) *bxd*-PRE, (D) *HGTX*-PRE, (E) *knrl*-PRE and control transcriptionally inactive intergenic region on (F) Chromosome 3L. Third instar larvae homozogous for the transgenes were used for ChIP. Consistent with the strong repression of the *white* reporter gene in all three cases the transgenic PREs (marked with asterisks) are immunoprecipitated as efficiently as the endogenous *bxd*-PRE (positive control) and much stronger than transgenic (control*) and endogenous control (Control) sites.

A number of PREs from our computationally defined list (41) have been tested in transgenic assays and all showed autonomous recruitment of PcG complexes and robust repression of associated reported genes (for the compendium of *Drosophila* PREs tested in transgenic assays, see (26)). However, all PREs tested so far have been of the PRC2:PRC1-dependent kind. We therefore asked whether computationally defined PREs that recruit PRC1 and PRC2 complexes independently in their native chromatin context can recruit both complexes when inserted elsewhere in the genome. For this purpose, we generated four constructs in which ∼1 kb DNA fragments centered on the *bxd*-PRE (well characterized PRC2:PRC1-dependent PRE of the *Ubx* gene), *HGTX*-PRE, *Knrl*-PRE (the two PRC2:PRC1-independent PREs from this study) and the intergenic site on chromosome 3L (the site used as a negative control in our ChIP-qPCR assays) were placed next to the *white* reporter gene, that confers red eye pigmentation. The *white* gene was separated from the PRE fragments by a *gypsy* insulator element flanked by FRT sites (Figure 3A). The constructs were integrated in the same 51C chromatin landing site by targeted ϕC31 *att* recombination (48). The 51C site is transcriptionally ‘inert’ (48) and does not by itself bind PcG proteins (32,41). Site-specific integration allowed direct comparison of the PRE properties within the same chromatin environment. As summarized in Figure 3B, the site-specific integration in flies lacking endogenous *white* function yields flies with orange eyes. The excision of the *gypsy* cassette from the control transgene leads to slight reduction in eye pigmentation suggesting that the chromatin environment of 51C site is by itself slightly repressive. Importantly, the excision of the *gypsy* cassette in all PRE containing transgenes yields flies with completely white eyes (Figure 3B). ChIP of transgenic PRE fragments, but not the transgenic control fragment, with antibodies against Pc and H3K27me3 strongly suggests that the repression is mediated by PcG mechanisms (Figure 3C–F). We conclude that a PRC2:PRC1-independent PRE can autonomously recruit PRC complexes and repress reporter genes.

When subjected to PcG repression the reporter *white* gene is often expressed in variegated fashion (57,58), especially when the transgene is in the homozygous state, due to somatic pairing of homologous chromosomes that enhances the repression (26,57). The complete repression of the *white* gene in our constructs integrated into the 51C site appears unusually strong. To ascertain that the ability of PRC2:PRC1-independent PREs to autonomously recruit PRC complexes is not limited to a fortuitously chosen permissive chromatin environment, we performed an additional set of transgenic experiments. We generated P-element constructs containing a ∼1.1 kb fragment spanning the presumptive PRC2:PRC1-independent PRE of the *Doc1* gene (*Doc1* transgenes, Supplementary Figure S6A) or, as a positive control, a ∼0.7 kb fragment spanning the presumptive PRC2:PRC1-dependent PRE of the *Doc3* gene (*Doc3* transgenes, Supplementary Figure S6A). The constructs contained the reporter *white* gene and the *lacZ* gene, not relevant for this study but used to screen for tissue-specific transcriptional enhancers within the *Doc* cluster (DS and IR, in preparation). The transgenic PRE fragments and the adjacent *lacZ* gene were shielded from the influence of outside chromatin environment by a pair of *gypsy* insulator elements (Supplementary Figure S6B).

In a *white* deficient but otherwise wild-type genetic background, flies heterozygous for the insertion of *Doc1* or *Doc3* transgenes have eye colors ranging from red to pale orange (Supplementary Figure S6C). This is expected as the transgenic *white* is shielded from the *Doc* PREs by the *gypsy* insulator element (Supplementary Figure S6B). When the *gypsy* insulator function is impaired by mutations in the *su(Hw)* gene (59), the eye color of the flies with one copy of the *Doc1 line 2* transgene becomes strongly variegated indicating strong repression of the transgenic *white* (Supplementary Figure S6C). The eye color of other heterozygous transgenic insertions does not show much change compared to that in wild-type background (Supplementary Figure S6C). Strikingly, however, when made homozygous, the eye color of all transgenic flies becomes highly variegated and the eyes of *Doc1 line 2* flies become completely white (Supplementary Figure S6C). These results argue that when the insulation by *gypsy* elements is lifted, the PRC2:PRC1-independent PRE of the *Doc1* gene, as well as the control PRC2:PRC1-dependent PRE of the *Doc3* gene, both produce efficient pairing-sensitive repression of the reporter gene.

The concomitant immunoprecipitations of transgenic PREs with antibodies against Pc and H3K27me3 strongly suggest that the repression is mediated by PcG mechanisms. Importantly, when the *gypsy* insulators are functional, the ChIP experiments show that Pc and H3K27me3 are associated with the transgenic PREs but not with the transgenic *white* gene (Supplementary Figures S6D–G). The latter argues that transgenic PREs can recruit Pc and H3K27me3 autonomously and that their immunoprecipitation is not due to an endogenous PcG target gene fortuitously near the insertion site. Consistently, inverse PCR mapping indicates that the transgenes are integrated far from endogenous PcG target genes (Supplementary Table S4). Overall, we conclude that PRC2:PRC1-independent PREs can autonomously recruit PRC complexes and repress reporter genes as efficiently as their PRC2:PRC1-dependent counterparts.

### H2A ubiquitylation does not explain the PRC2:PRC1 dependence

**Figure 4.**
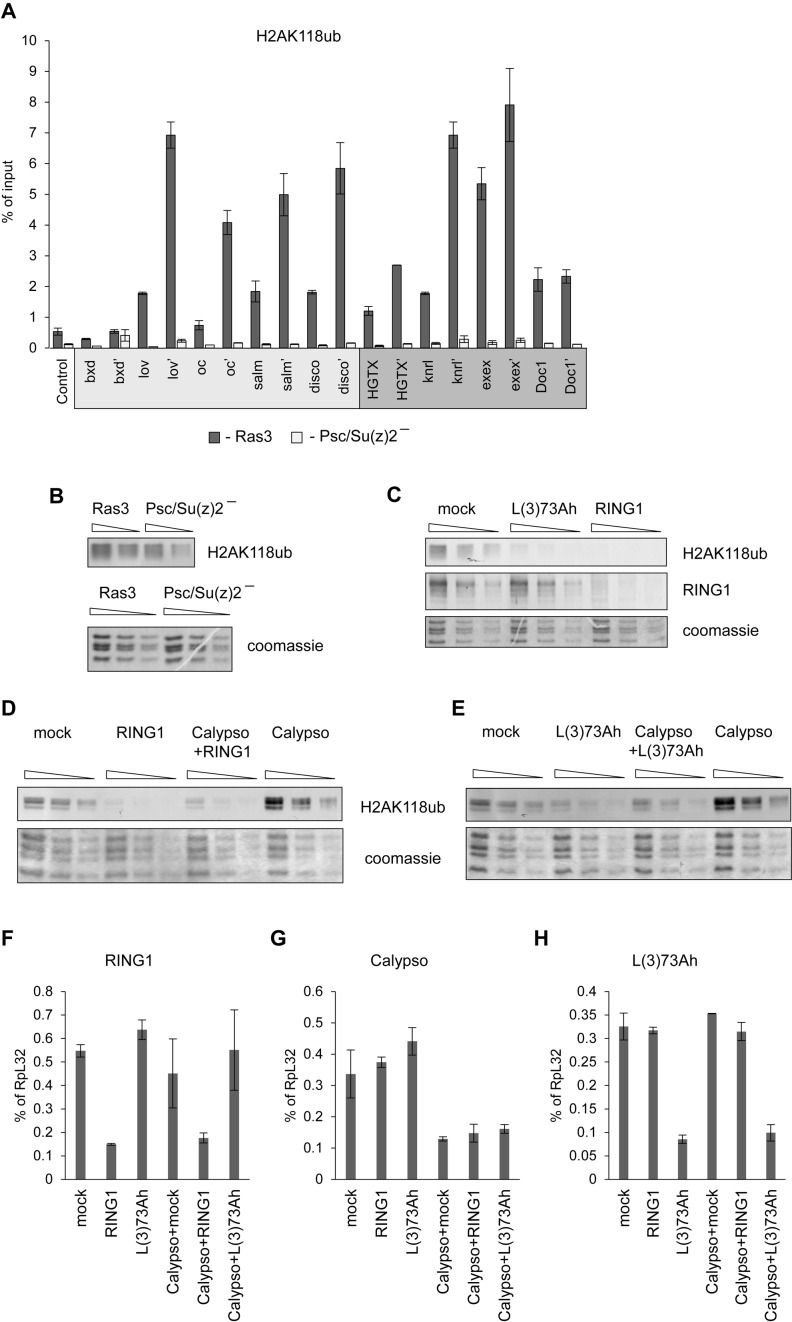
Contribution of *Drosophila* RING1-PCGF complexes to H2AK118 ubiquitylation. (**A**) ChIP-qPCR analyses of H2AK118ub at PRC2:PRC1 dependent (light grey box) and of PRC2:PRC1 independent (dark grey box) PREs in control Ras3 cells and Psc/Su(z)2 deficient cells. Pairs of amplicons correspond to PRE-cores and PRE flanks (marked with apostrophe). (**B**) Two-fold dilutions of histones acid-extracted from Ras3 and Psc/Su(z)2 minus cells were analyzed by Western-blot with antibodies against H2AK118ub. Coomassie stained SDS-PAGE gels of the histone samples (below) were used as loading control. (**C–E**) Two-fold dilutions of total nuclear protein from S2 cells subjected to knock-down with dsRNAs indicated above the corresponding lanes were analyzed by Western-blot with antibodies against H2AK118ub and RING1 or coomassie staining. Note, that the knock-down of L(3)73Ah protein leads to dramatic reduction of H2AK118ub level and suppresses the increase of H2AK118ub following Calypso knock-down. (**F–H**) RT-qPCR assay of RNAi knock-down efficiency. Transcript abundance is normalized to that of the housekeeping *RpL32* gene. The bars indicate the mean of two independent experiments and whiskers show the scatter. Combinations of dsRNAs used for corresponding knock-down experiments are shown over x-axes.

Recent reports indicate that in mouse cells the PRC2 subunit AEBP2 can directly interact with histone H2A monoubiquitylated at Lysine K119 (H2AK119ub) and that H2AK119ub is sufficient to trigger the recruitment of PRC2 (22,28,33). We therefore asked whether the steady state levels of H2AK118ub (the fly analog of mammalian H2AK119ub) within and around PREs correlate with PRC2:PRC1 dependence. ChIP-qPCR analysis of selected PREs shows no correlation between H2AK118ub levels and PRC2:PRC1 dependence (Figure 4A). Importantly, it also shows that at both PRC2:PRC1-dependent and PRC2:PRC1-independent PREs H2AK118ub is completely lost in cells where Psc/Su(z)2 is absent. The latter excludes the possibility that at PRC2:PRC1-independent PREs H2AK118ub is produced by a RING1 complex with PCGF subunit other than Psc or Su(z)2. We also note, that PRC2:PRC1-dependent *bxd* PRE has virtually no H2AK118ub either in wild type or in Psc/Su(z)2 deficient cells. Taken together these results indicate that in *Drosophila* H2AK118ub is not generally necessary for PRC2 recruitment.

Despite dramatic loss of H2AK118ub within and around PREs the overall H2AK118ub level in Psc/Su(z)2 minus cells remains at 60–70% of that in the wild-type cells (Figure 4B). The *Drosophila* genome encodes three PCGF proteins: Psc, Su(z)2 and L(3)73Ah. The latter is an ortholog of mammalian PCGF3. The RNAi knock-down of L(3)73Ah in Schneider 2 (S2) cells leads to over 70% reduction of the overall H2AK118ub (Figure 4C) while the knock-down of RING1 protein brings H2AK118ub below detection limit (Figure 4C). Taken together these results suggest that in cultured *Drosophila* cells RING1-L(3)73Ah complexes are responsible for the bulk of the steady state H2AK118ub, most of which must be outside PcG target regions. Consistently, the recent genome-wide mapping of *Drosophila* H2AK118ub indicates that this modification is wide-spread and in addition to PcG targets is also highly enriched at sites with no enrichment of H3K27me3 or PcG proteins (10,60).

The steady state level of H2AK118ub is controlled by two opposing activities: ubiquitylation by RING1-containing complexes and de-ubiquitylation by the PR-DUB complex (37). We therefore considered the possibility that although the bulk of H2AK118ub in the steady state is the product of a RING1-L(3)73Ah complex, the Psc/Su(z)2-RING1 complexes at PcG target genes might actually produce much more H2AK118ub but this ubiquitylation is specifically removed by PR-DUB. The RNAi knock-down experiments argue that this is not the case. Consistent with previous reports (37) the knock-down of Calypso, the ubiquitin C-terminal hydrolase subunit of PR-DUB, leads to ∼8-fold increase in the overall level of H2AK118ub (Figure 4D). As expected, this increase in H2AK118ub is abolished by simultaneous knock-down of Calypso and RING1 (Figure 4D). Importantly, the effect of Calypso knock-down is also completely suppressed by simultaneous knock-down of L(3)73Ah (Figure 4E). The latter indicates that L(3)73Ah-dependent H2AK118 ubiquitylation outside PcG target genes is, in fact, the primary target of PR-DUB activity. That said, our experiments with Psc/Su(z)2 minus cells suggest that three quarters of RING1 molecules are in a complex with Psc or Su(z)2 and are degraded in their absence (Figure 1B). Consistently, RNAi knock-down of L(3)73Ah results in no detectable change in the total amount of RING1 (Figure 4C). This suggests, somewhat surprisingly, that the majority of H2AK118 ubiquitylation is done by the minority of RING1 complexes. The latter fits well with observations that mammalian RING-PCGF-RYBP complexes are more potent H2A ubiquitylases than PRC1 (22).

### PRC1 binding to PREs is not strictly dependent on PRC2 and H3K27 methylation

**Figure 5.**
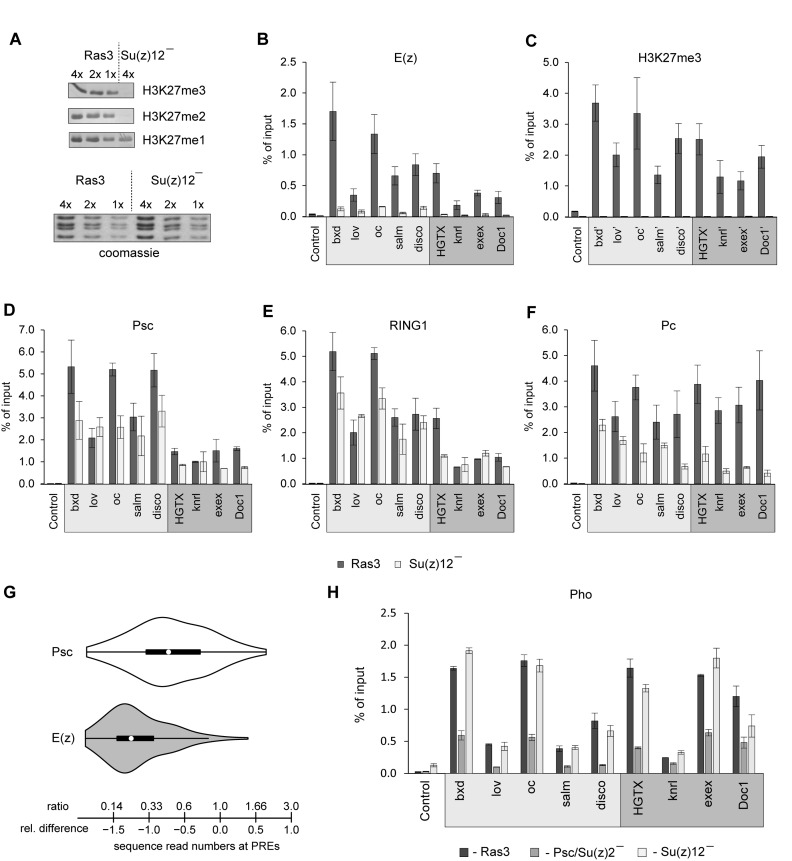
H3K27me3 is not essential for PRC1 binding to PREs. (**A**) Indicated amounts of histones acid-extracted from Ras3 and Su(z)12 minus cells were analyzed by Western-blot with antibodies against H3K27 methylated to various degrees. Coomassie staining (below) was used to control the loading. (**B–F**) ChIP-qPCR analyses of PcG proteins and H3K27me3 at PRC2:PRC1 dependent (light grey box) and PRC2:PRC1 independent (dark grey box) PREs in control Ras3 cells and PRC2 deficient Su(z)12 minus cells. (**G**) Comparison of violin plots of the changes in the number of E(z) ChIP-seq reads at PREs between Psc/Su(z)2 deficient and Ras3 control cells (grey plot) and Psc ChIP-seq reads between Su(z)12 minus and Ras3 control cells (white plot). Violin plots combine box plots with observation density plots. The black boxplots span the inter-quartile range. The whiskers indicate the adjacent values plus or minus 1.5 time inter-quartile range. The white circles mark positions of the medians. The scale indicates the changes in sequence read numbers expressed as relative differences (below) or ratios (above). The E(z) violin plot is skewed to the left and has long right tail, which indicates that the majority of the PREs lose the signal when PRC1 is absent but there is a class of PREs that shows little change. In contrast, the violin plot for Psc is symmetrical and shows no evidence for the two classes of PREs with different dependence on PRC2. These observations are robust as we obtained essentially the same results using a PRE set defined from genomic binding profiles of Psc, Pc and E(z) in Ras3 cells (Supplementary Figure S8, Supplementary Table S5). (**H**) ChIP-qPCR analyses of the Pho protein at PRC2:PRC1 dependent (light grey box) and PRC2:PRC1 independent (dark grey box) PREs in control (Ras3), Psc/Su(z)2 and Su(z)12 deficient cells.

The role of H3K27me3 in coordinating the recruitment of PcG complexes has been controversial. To address this question we turned to cultured cells derived from *Su(z)12^4^* mutant embryos. Here, we present in detail experiments done with *S12-26I3* cell line, hereafter referred as Su(z)12 minus cells. However, we obtained essentially the same results with cells from the independently derived *S12-27IIb* line, indicating that the effects are linked to *Su(z)12^4^* mutation and not to a particular cell isolate. In Su(z)12 minus cells, no Su(z)12 protein is produced, which leads to degradation of the E(z) protein and complete loss of di-and tri-methylated H3K27 (Figures 1 and 5A). The loss of PRC2 does not affect the overall levels of PRC1 components or the bulk H2AK118ub (Figure 1, Supplementary Figure S7A). Remarkably, ChIP-qPCR analysis shows that in the mutant cells, complete loss of H3K27me3 and dramatic reduction of E(z) and Pcl ChIP-signals (Figure 5B and C, Supplementary Figure S7B) are accompanied by a very modest (<2-fold) reduction of immunoprecipitation with antibodies against Psc and RING1 (Figure 5D and E). This is seen at all representative PREs irrespective of their PRC2:PRC1 dependence. The immunoprecipitation with antibodies against Pc is more affected by the PRC2 and H3K27me3 loss. Nevertheless in all cases the Pc ChIP signals in Su(z)12 deficient cells remain significantly higher than those in the *Psc/Su(z)2* minus cells lacking PRC1 (compare Figure 1D and Figure 5F). Taken together these observations suggest that a significant fraction of PRC1 is recruited to PREs of both kinds independently of PRC2 and H3K27me3.

To test this conclusion further, we compared the binding of the Psc subunit of PRC1 at PREs in Su(z)12 minus and control Ras3 cells using ChIP-seq. We did two independent ChIP-seq experiments for each genetic background and also had the DNA from corresponding chromatin input materials sequenced. Corroborating the ChIP-qPCR results, the ChIP-seq analyses show ∼2-fold reduction of the Psc ChIP-seq signal at most PREs in the mutant cells (Figure 5G). Compared to the changes in E(z) ChIP-seq signals in Psc/Su(z)2 minus cells, the loss of Psc ChIP-seq signals in Su(z)12 minus cells is significantly smaller (median Psc reads ratio = 0.47; median E(z) reads ratio = 0.23; p = 3.745e-13, Wilcoxon rank sum test). Importantly, in contrast to the distribution of changes of E(z) signals, which is skewed to the left and has a long right tail, the distribution of changes of Psc signals is nearly symmetrical about the median. The cases of extreme Psc signal loss are few and may represent genes that are not PcG-repressed in this particular cell line since the repertoire of genes repressed by PcG mechanisms varies within a 10% range among different cultured wild-type cell lines (41). Overall, we conclude that PRC1 can be targeted to PREs in the total absence of PRC2 and H3K27 tri-methylation. We find no evidence of a special class of PREs where the recruitment of PRC1 strictly requires PRC2.

### PRC1–PhoRC connection

PhoRC is an auxiliary protein complex that is essential for the repression of many *Drosophila* PcG target genes (54,61). How PhoRC contributes to PcG repression is not very clear, but one among several proposed mechanisms envisions that it interacts directly with PRC2 and helps to anchor it to PREs (29). We and others have shown that in addition to sequence-specific interaction of its Pho subunit with the DNA, the efficient binding of PhoRC to PREs requires PRC1 (45,62,63). We, therefore, wondered whether the loss of PRC2 from PREs in the PRC1 deficient cells is due to the destabilization of PhoRC binding and whether the main difference between PRC2:PRC1-dependent and independent PREs is in how well they bind Pho when PRC1 is absent. To address this question we performed ChIP with antibodies against Pho and chromatin from Psc/Su(z)2 minus, Su(z)12 minus and control Ras3 cells and analyzed the results by qPCR at our panel of PREs. As summarized in Figure 5H, the loss of PRC1 leads to the reduction of immunoprecipitation with anti-Pho antibodies at all PREs regardless of their PRC2:PRC1-dependence. In contrast, the loss of PRC2 and H3K27 methylation has no effect on Pho ChIP-qPCR signals. We conclude that the cross-talk between PRC1 and PhoRC does not involve PRC2 and that the difference in PRC2:PRC1-dependence is not explained by the ability of some PREs to bind PhoRC efficiently even when PRC1 is absent.

### H3K27me3 helps PcG complexes anchored at PREs to interact with surrounding chromatin

**Figure 6.**
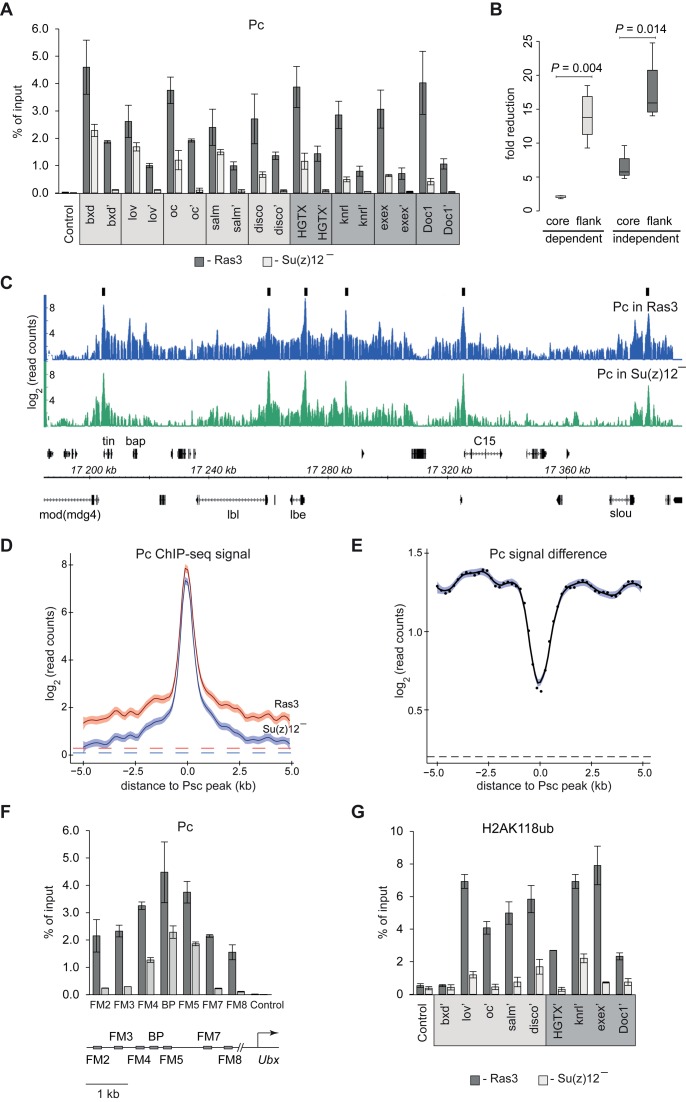
Loss of H3K27me3 makes interaction of Pc with chromatin around PREs less stable. (**A**) ChIP-qPCR analysis shows that in Su(z)12 minus cells the immunoprecipitation of chromatin with antibodies against Pc is preferentially lost at amplicons flanking PREs (marked with apostrophes) compared to amplicons corresponding to PRE cores. Here and in (G), PRC2:PRC1 dependent PREs are marked with light grey box and PRC2:PRC1 independent PREs are marked with dark grey box. (**B**) The fold reduction of Pc ChIP signals at core and flanking amplicons at PRC2:PRC1 dependent (light grey) and PRC2:PRC1 independent (dark grey) PREs is summarized in box-plots. The boxplots indicate the median and span the inter-quartile range. The whiskers indicate the lowest and the highest values. The differences between PRE cores and flanks are statistically significant (Wilcoxon rank sum test). (**C**) ChIP-seq profiles of Pc binding within the cluster of NK-homeobox genes in Ras3 and Su(z)12 minus cells. Note that in the mutant cells the signal at Pc ‘tails’ is reduced more than at peak summits. The positions and the exon structure of annotated transcripts are shown above (transcribed left to right) and below (transcribed right to left) the coordinate scale (in kb). Black boxes illustrate positions of Psc peaks used in the analyses below. (**D**) Cumulative distributions of Pc ChIP-seq signals around 122 isolated (>10 kb to the nearest neighbor) Psc peaks. The shades around fitted curves indicate 95% confidence interval. Dashed lines indicate average ChIP-seq signals outside PcG target genes in Ras3 (red) Su(z)12 deficient cells (blue). (**E**) The difference between cumulative distributions of ChIP reads, but not of the reads from corresponding input materials (Figure S9B and C), illustrates that the ChIP-seq signal at a distance from the Psc peaks is reduced more than at peak summits. Black dots indicate the differences between corresponding points on fitted curves in D and the blue shade indicates the 95% confidence interval for fitting the smooth curve. The difference between average signals outside PcG target genes (black dashed line) is shown for comparison. (**F**) Detailed analysis of Pc ChIP around *bxd*-PRE in control Ras3 and Su(z)12 minus cells. The relative position of amplicons is shown below the histogram. ‘BP’ amplicon marks the *bxd*-PRE core (30). (**G**) The results of ChIP-qPCR with antibodies against H2AK118ub.

PcG complexes anchored to PREs loop out and interact with surrounding chromatin (64). This looping can be blocked by chromatin insulator elements (64) and is responsible for the low-level crosslinking of Pc but not of the other PRC1 components at extended distances from PREs (30–32,64,65). Also in this case, the low-level crosslinking is thought to reflect the direct interaction between nucleosomes harboring H3K27me3 and the chromodomain of the Pc protein. This model predicts that the loss of H3K27me3 should preferentially reduce the crosslinking and ChIP of Pc at a distance from PREs. In striking agreement with this prediction, we see that in contrast to 2-fold reduction of Pc ChIP signals at the cores of PRC2:PRC1-dependent PREs the ChIP signals in their vicinity are reduced 9- to 18-fold, virtually to background level (Figure 6A and B, see also Supplementary Figure S2 for positions of amplicons). Even at PRC2:PRC1-independent PREs, where Pc ChIP signals at the PRE cores appear to be more affected by the loss of PRC2, the loss of ChIP signals at a distance is significantly stronger (Figure 6A and B). Genome-wide mapping of products from replicate Pc ChIP experiments in Su(z)12 deficient and control Ras3 cells further corroborates our findings. First, it indicates that the loss of H3K27 methylation does not cause massive re-distribution of major PRC1 binding sites (Supplementary Figure S9A). Second, as illustrated by the case of the NK-homeobox gene cluster (Figure 6C) and by comparison of cumulative distributions of Pc ChIP-seq signals around high-confidence Psc peaks (Figure 6D and E, Supplementary Figure S9B and C), it is clear that preferential loss of Pc immunoprecipitation at a distance from high-affinity binding sites is a genome-wide effect.

The detailed analysis of the ChIP in the immediate vicinity of the *bxd*-PRE shows that the preferential loss of Pc ChIP signal occurs at a range as short as 1 kb from the PRE core (Figure 6F). The latter strengthens the idea that the role of H3K27me3 is not to increase the frequency of the looping contacts, which are always high at short distances (66), but to make the contacts more stable. We speculate that the stabilized interaction of PcG complexes with the chromatin of target genes is critical for their repressive function. Although we have yet to fully understand what aspect of repression requires stable interactions, we see that in the absence of H3K27me3 the levels of H2AK118 ubiquitylation around PREs are reduced (Figure 6G).

## DISCUSSION

At most PcG target genes, PRC1 and PRC2 act in concert and both complexes must be targeted to a gene to achieve robust PcG repression. Because PRC1 can specifically recognize the H3K27me3 modification placed by PRC2 (27) and PRC2 can specifically recognize the H2AK118ub placed by PRC1 (28), the two histone modifications have been viewed as key factors to coordinate PRC1 and PRC2 recruitment (22,29). Our observations challenge this view in several ways. First, we show that a significant fraction of PRC1 binds PREs in the complete absence of H3K27me3, indicating that this histone modification is not essential for PRC1 targeting to PREs. Second, we demonstrate that there is no strict correlation between the binding of PRC2 and the presence of H2AK118ub and roughly one-third of *Drosophila* PREs recruit PRC2 independently of H2AK118ub. Finally, we show that PREs differ in the way they recruit PcG complexes, suggesting that there is no unique hierarchy for coordinated recruitment of PRC1 and PRC2. In addition to PREs, PRC1 was reported to bind promoters of many active genes (67). This binding is at least an order of magnitude weaker than that at PREs and is not accompanied by the presence of PRC2 and H3K27me3 (67,68). These promoter–proximal PRC1 binding sites have not been considered in the current study.

### The interdependence between PRC1 and PRC2

Another key finding of our work is that at approximately two-thirds of PREs the binding of PRC2 requires the presence of PRC1 and/or dRAF complexes. Recently, RING2-KDM2B complexes (also referred to as non-canonical PRC1 or PRC1.1) were shown to drive the recruitment of PRC2 complexes in mouse embryonic stem (ES) cells (22,33). However, in that case the underlying mechanism appears to be different. In mouse ES cells, the ubiquitylation of H2AK119 is required to mediate PRC2 recruitment (22,33,69) while it does not explain the PRC2:PRC1 dependence in flies. In fact, we find that the bulk of *Drosophila* H2AK118ub is produced by the RING1-L(3)73Ah complexes most likely outside of the PcG target genes. Flies with mutations in the *l(3)73Ah* gene die at late larval or pupal stages and show no homeotic transformations (70). This reinforces the idea that the RING1-L(3)73Ah complexes and the bulk H2AK118ub have little relation to PcG repression. Consistent with our findings, the analysis of flies with point mutations in the H2A residues ubiquitylated by RING1 complexes indicates that H2A ubiquitylation is not required for PcG repression in *Drosophila* (36). Interestingly, our data indicate that the bulk H2AK118ub is actively removed by the PR-DUB deubiquitylase activity. Thus, although the most obvious phenotypic effects of the mutations in genes encoding *Drosophila* PR-DUB subunits highlight its involvement in the repression of the *HOX* genes (37), the main function of PR-DUB may lie outside of PcG regulation. Considering that the analogous mammalian complex has very broad genomic binding (71), this previously unappreciated role may be the one that is evolutionary conserved.

How *Drosophila* PRC1 and/or dRAF promote PRC2 recruitment remains an open question. One attractive possibility is that PRC1 or dRAF directly interact with PRC2. Supporting this hypothesis PRC1 and the Esc subunit of PRC2 were reported to co-immunoprecipitate from the nuclear extracts made from early *Drosophila* embryos (72). The co-immunoprecipitation was not detected at later stages of development but the interaction may still occur specifically at PREs thereby promoting PRC2 binding even at later developmental stages. Interestingly, the human EED protein was also reported to interact with PRC1 in several human and mouse cultured cells (73), suggesting that potential Esc/EED-mediated cross-talk between the two kinds of PRC complexes may be evolutionary conserved. Another possible link between PRC1 and PRC2 may involve the Scm protein. Scm was originally co-purified as a substoichiometric component of PRC1 by fractionation and conventional affinity chromatography (14). However, in recent experiments that used formaldehyde crosslinking prior to affinity purification, Scm was also recovered as an interactor of PRC2 (74).

### The role of H3K27 methylation

Substitution of histone H3 genes with a mutant variant encoding unmethylatable Alanine instead of Lysine at position 27 phenocopies loss-of-function mutations in PRC2-encoding genes (6). This indicates that H3K27 is the PRC2 substrate relevant for PcG repression and that H3K27 methylation is essential for the process. Here, we show that PRC1 is still recruited to PREs when H3K27me3 is entirely absent, which contradicts the model that places PRC2 at the base of the recruitment hierarchy (29). Instead we find that one role of PRC2-catalyzed H3K27me3 is to stabilize the interaction of PRE-anchored PcG complexes with surrounding chromatin that harbors enhancers, promoters and transcription units of target genes. We speculate that prolonged interactions with these gene regions are an integral part of repression that may act by inhibiting chromatin remodeling (14), interfering with transcription initiation (75) or elongation (76) or suppressing transcriptional enhancers (77). The drastic reduction of H2AK118ub around PREs in the absence of PRC2/H3K27me3 is likely due to the less stable interaction of the PRE-anchored PRC1 with surrounding chromatin. The stable loops between PREs and surrounding chromatin may also help PRC2 to spread and maintain the high level of H3K27me3 over considerable distances from its anchor points, thereby reinforcing the system. Finally, the stable and exhaustive tri-methylation of H3K27 may contribute to the repression directly by competing with acetylation of H3K27 by the general transcriptional co-activator CBP (78,79).

### Implications for plasticity of PcG repression

PcG proteins are present in all cells and most of the PcG target genes are repressed in a given cell type (41,80,81). Nevertheless each cell has a subset of target genes that remain active to define its developmental identity. How does the PcG system combine the ability to repress target genes in an epigenetically stable way with the plasticity to allow selected target genes to be expressed when needed?

As follows from our observations, abolishing H3K27me3 within a promoter or transcriptional unit of a target gene will impair the interactions of PRE-anchored PcG complexes with these gene elements, which, as we propose, will impair the repression. H3K27me3 can be removed by histone de-methylation (10,82,83) or histone H3 replacement aided by acetylation of H3K27, all of which are mechanistically linked to transcriptional activity. Consistently, during embryonic and larval development, cells expressing *Drosophila HOX* genes retain PcG proteins at corresponding PREs but lose H3K27me3 from the surrounding chromatin (31,84). We suspect that the relief from PcG repression mediated by the removal of H3K27me3 is contingent on the ongoing transcription and quickly lost when transcriptional activators are no longer available.

A more radical way to prevent the repression is to displace PcG complexes from PREs and such examples have also been reported (41,55). What triggers the displacement of PcG complexes as opposed to simple loss of H3K27me3 is not clear, but Trithorax Group proteins are likely to be involved. How difficult it is to displace PcG complexes from a given PRE depends on a number and kind of cooperative interactions used to recruit them. We envision, that two different ways to recruit PRC2 contributing to different extents at different PREs would fine-tune the robustness of PcG repression in a locus-specific manner. It will be interesting to test whether PRC2:PRC1 independent PREs are more or less robust in retaining PcG complexes compared to their PRC2:PRC1 dependent counterparts. Overall, we propose that the use of multiple cooperative interactions to recruit PcG complexes to PREs and of H3K27me3 to ‘pass’ the repressive activity from PREs to target genes are integral for epigenetic plasticity of Polycomb group regulation.

## Supplementary Material

SUPPLEMENTARY DATA

## DATA ACCESS

The ChIP-seq data are available from GEO at the accession number GSE83248.
