# Supplementary material for: Interdependence of PRC1 and PRC2 for recruitment to Polycomb Response Elements
Source: Nucleic Acids Res. 2016 Aug 23;44(21):10132–49. doi: 10.1093/nar/gkw701 (PMC5137424; doi:10.1093/nar/gkw701)
Supplement: SUPPLEMENTARY DATA [file supp_44_21_10132__index.html]

Interdependence of PRC1 and PRC2 for recruitment to Polycomb Response Elements — Interdependence of PRC1 and PRC2 for recruitment to Polycomb Response Elements — SUPPLEMENTARY DATA 

# Interdependence of PRC1 and PRC2 for recruitment to Polycomb Response Elements

## SUPPLEMENTARY DATA

- SUPPLEMENTARY DATA
- SUPPLEMENTARY DATA
