# Supplementary material for: Interdependence of PRC1 and PRC2 for recruitment to Polycomb Response Elements
Source: Nucleic Acids Res. 2016 Aug 23;44(21):10132–49. doi: 10.1093/nar/gkw701 (PMC5137424; doi:10.1093/nar/gkw701)
Supplement: SUPPLEMENTARY DATA [file supp_gkw701_nar-00469-x-2016-File008.pdf]

Interdependence of PRC1 and PRC2 for recruitment to Polycomb Response Elements

Tatyana G. Kahn[1], Eshagh Dorafshan[1], Dorothea Schultheis[2], Aman Zare[1], Per Stenberg[1,3], Ingolf Reim[2], Vincenzo Pirrotta[4] and Yuri B. Schwartz[1#]

1. Department of Molecular Biology, Umeå University, Umeå, Sweden
2. Friedrich-Alexander University of Erlangen-Nürnberg, Department of Biology, Division of Developmental Biology, Erlangen, Germany
3. Division of CBRN Defense and Security, Swedish Defense Research Agency, FOI, Umeå, Sweden
4. Department of Molecular Biology and Biochemistry, Rutgers University, Piscataway, New Jersey, USA

# Correspondence should be addressed to: yuri.schwartz@umu.se

Supplementary materials.

1. Supplementary figure legends
2. Nine supplementary figures
3. Five supplementary tables

Supplementary figure legends

Figure S1. Ablation of PRC1 or PRC2 does not lead to major changes in expression of PcG genes. RT-qPCR analyses indicate that there is no major difference in the expression of PcG genes in different cultured cell lines. Most importantly the differences in gene expression do not correlate with major effects on the corresponding protein levels (compare to Figure 1B). The bars indicate the mean of two independent experiments and whiskers show the scatter.

Figure S2. Schematic of PcG target genes selected for ChIP-qPCR analyses. Gene exons are shown as grey boxes, the introns are indicated with broken lines and the adjacent genomic DNA is shown as straight lines. The schematics are drawn to different scales indicated with bars above each gene. The positions of PCR amplicons used for the analyses are marked with numbered arrows. The arrows labeled with "1" correspond to amplicons from PRE cores, the arrows labeled with "2" mark the amplicons to assay H3K27me3 and H2AK118ub. **A**. Genes with PRC2:PRC1 dependent PREs. **B**. Genes with PRC2:PRC1 independent PREs. Note the two alternative TSS of *lov* gene.

Figure S3. PRC1 ablation does not lead to strong transcription thorough PREs, ASH1 binding or general strong activation of PcG target genes. A. The transcription through PREs was assayed by RT-qPCR. Here and in **B** and **C** the histograms show the mean of two independent experiments with error bars indicating the scatter. The NC (Negative Control) values are for a short amplicon from the intergenic region of Chromosome 3L and indicate the detection threshold for the assay. **B.** Expression of selected PcG target genes. **C.** The binding of ASH1 to PREs as assayed by ChIP-qPCR. The PC (Positive Control) values are for a short amplicon from transcriptionally active PcG target gene *pointed* (*pnt*).

Figure S4. The ablation of PRC1 does not affect untargeted di-methylation of H3K27 by PRC2. A. Two-fold dilutions of acid-extracted histones from control Ras3 and Psc/Su(z)2 mutant cell lines were analyzed by western-blot with antibodies against H3K27me2 and H3K27me3. Note that the ablation of Psc/Su(z)2 has no significant effect on the overall H3K27me2/me3, which suggests that much of the bulk H3K27me3 is produced by untargeted PRC2 activity and that the integrity and the activity of PRC2 *per se* do not require PRC1. **B.** H3K27me2 in the vicinity of PREs as measured by ChIP-qPCR. Histograms show the mean of two independent experiments with error bars indicating the scatter. Note that in PRC1 deficient cells the loss of H3K27me3 from the vicinity of PRC2:PRC1 dependent PREs (light gray box) leads to di-methylation of H3K27 by untargeted PRC2. In contrast, since the levels of H3K27me3 around PRC2:PRC1 independent PREs (dark grey box) in PRC1 deficient cells remain high no H3K27me2 is added. The high levels of H3K27me2 at "Control" amplicon from transcriptionally inactive intergenic region on Chromosome 3L are similar in *Ras3* and Psc/Su(z)2 minus cells.

Figure S5. Inhibition of transcriptional elongation has no detectable effect on E(z) binding at PREs. A. Nuclear extracts from DRB- or mock-treated Psc/Su(z)2 minus cells were analyzed by western-blot with antibodies against hypophosphorylated isoform of RNA Polymerase II (RNAPII) or RNA Polymerase II phosphorylated at Serine 2 (RNAPII Ser2-P). Note that the DRB treatment leads to ~10-fold reduction of the RNAPII Ser2-P and increases the level of unposphorylated RNAPII. **B.** Coomassie stained SDS-PAGE gels of the corresponding nuclear extracts were used as loading control. **C**. RT-qPCR analysis of unspliced RNAs from house-keeping *Taf4* gene and derepressed PcG targets *oc* and *srp* genes indicates that DRB treatment inhibits transcription. Blue bars indicate the number of cDNA molecules in samples from DRB treated Psc/Su(z)2 minus cells and green bars show the number of cDNA molecules in mock-treated control. The copy numbers were derived by

comparing the Ct values in the RT-qPCR reactions to those in reactions with serial dilution of genomic DNA of known concentration. Here and in **D** the histograms show the mean of two independent experiments with error bars indicating the scatter. **D**. Despite dramatic reduction of RNAPII Ser2-P and inhibition of transcription the ChIP-qPCR signals for E(z) at PRC2:PRC1-dependent *bxd*-PRE or PRC2:PRC1-independent *HGTX*-PRE remain essentially the same in DRB and mock-treated cells.

Figure S6. Functional properties of PRC2:PRC1 independent PRE. A. ChIP-seq profiles of E(z) binding within *Doc* cluster in Ras3 and Psc/Su(z)2 deficient cells. The binding of E(z) to the PRE upstream of the *Doc1* gene is not affected by PRC1 ablation while the binding to the PRE of the *Doc3* gene is strongly reduced. The positions and the exon structure of annotated transcripts are shown above (transcribed left to right) and below (transcribed right to left) the coordinate scale (in bp). The positions of fragments used for transgenic assays are indicated with colored boxes. **B**. The *Doc* transgenic constructs are shown schematically approximately to scale with P-element ends (arrows), *gypsy* insulator elements (G), Doc genomic fragment (grey box), lacZ and mini-*white* genes indicated. The positions of PCR amplicons used for ChIP analysis in **D**.-**G**. are indicated with black lines. **C**. Expression of *white* gene in two independent *Doc1* and three independent *Doc3* transgenic lines. The eye color of male and female flies with one or two copies of transgene are shown in wild-type and *su(Hw)* minus backgrounds. The results for ChIP-qPCR analyses of Pc and H3K27me3 within *Doc1* (**D.-E.**) and *Doc3* (**F.-G.**) transgenic constructs. Third instar larvae homozygous for the transgenic insertions on wild-type background were used for ChIP. Consistently, Doc1* and Doc3* amplicons are immunoprecipitated at an extent comparable to that of the endogenous *bxd*-PRE (bxd and bxd' amplicons). However, since the *white* gene is shielded from PREs by functional *gypsy* insulator w* amplicon is not immunoprecipitated.

Figure S7. Loss of Su(z)12 has no effect on bulk H2AK118ub but impairs the binding of Pcl to PREs. A. Two-fold dilutions of histones acid-extracted from Ras3 and Su(z)12 minus cells were analyzed by western-blot with antibodies against H2AK118ub. Coomassie stained SDS-PAGE gel of the histone samples (below) was used as loading control. **B.** ChIP-qPCR analyses of Pcl at PRC2:PRC1 dependent (light grey box) and PRC2:PRC1 independent (dark grey box) PREs in control Ras3 cells and PRC2 deficient Su(z)12 minus cells.

Figure S8. Comparison of violin plots of the changes in the number of E(z) and Psc ChIP-seq reads at PREs defined from PcG biding profiles in Ras3 cells. PREs (n=211) were defined as Psc-bound regions overlapped by Pc- and E(z)-bound regions. Significantly bound regions were defined as clusters of coordinates that are no more than 500bp apart and whose values exceed 2.5 Standard Deviations of genomic mean. Note that 97% of our main PRE set is contained in the PRE set defined from Ras3 profiles. Grey plot indicates the changes in the number of E(z) ChIP-seq reads between Psc/Su(z)2 deficient and Ras3 control cells. White plot indicates the changes in the number of Psc ChIP-seq reads between Su(z)12 minus and Ras3 control cells. The black boxplots span the inter-quartile range. The whiskers indicate the adjacent values plus or minus 1.5 time inter-quartile range. The white circles mark positions of the medians. The scale indicates the changes in sequence read numbers expressed as relative differences (below) or ratios (above). The E(z) violin plot is skewed to the left and has long right tail, which indicates that the majority of the PREs lose the signal when PRC1 is absent but there is a class of PREs that shows little change. The violin plot for Psc is symmetrical and shows no evidence for the two classes of PREs with different dependence on PRC2.

Figure S9. Loss of Su(z)12 and H3K27me3 does not lead to the re-distribution of major PRC1 binding sites or systematic changes in sequencing assay. A. ChIP-seq profiles of Pc and Psc binding over Chromosome 2L in Ras3 and Su(z)12 minus cells. Note that the pattern

of major ChIP-seq peaks in the two cell lines is very similar. **B.** Cumulative distributions of sequencing reads in chromatin inputs around 122 isolated (>10kb to the nearest neighbor) Psc peaks. The shades around fitted curves indicate 95% confidence interval. Regions underneath Psc peaks are slightly overrepresented in the Input libraries from both cell types. However, the height of the "Input" peak is ~ 30 (2^5) times lower than that of the Pc ChIP-seq peak in Figure 6D. **C.** Importantly, there is little difference between cumulative distribution of Input signals from Ras3 and Su(z)12 minus cells. Black dots indicate the differences between corresponding points on fitted curves in **B** and the blue shade indicate the 95% confidence interval for fitting the smooth curve.

Figure S1

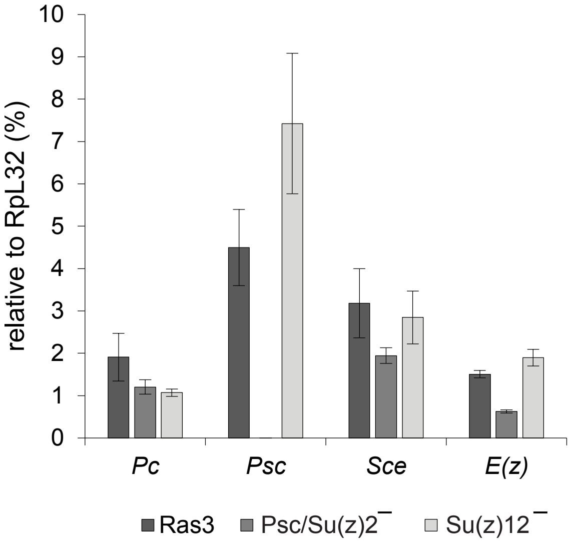

10
9
8
7
6
5
4
3
2
1
0
relative to RpL32 (%)
Pc
Psc
Sce
E(z)
Ras3
Psc/Su(z)2⁻
Su(z)12⁻

Figure S2

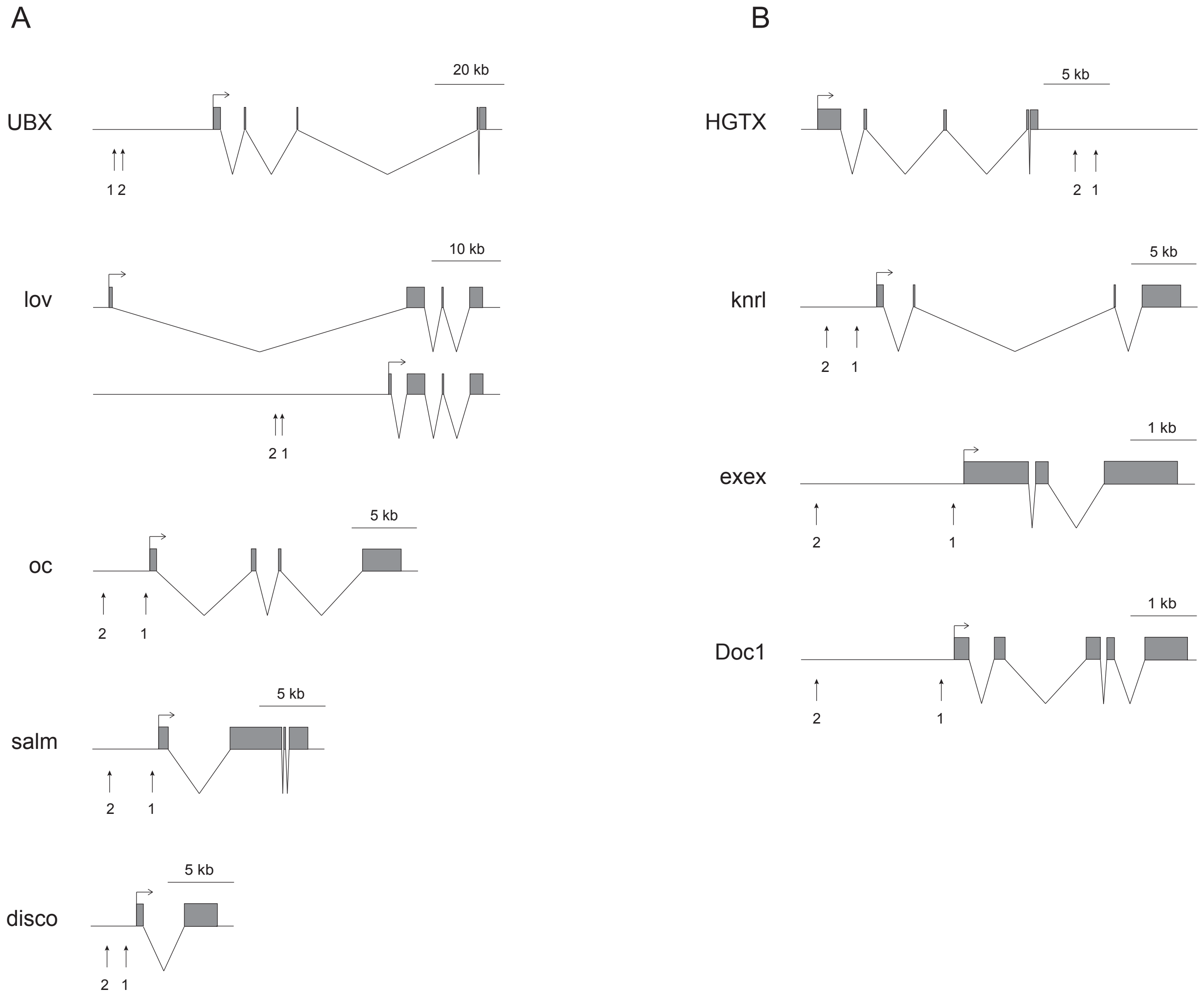
A
B
UBX
20 kb
1 2
lov
10 kb
2 1
oc
5 kb
2
1
salm
5 kb
2
1
disco
5 kb
2
1
HGTX
5 kb
2
1
knrl
5 kb
2
1
exex
1 kb
2
1
Doc1
1 kb
2
1

Figure S3

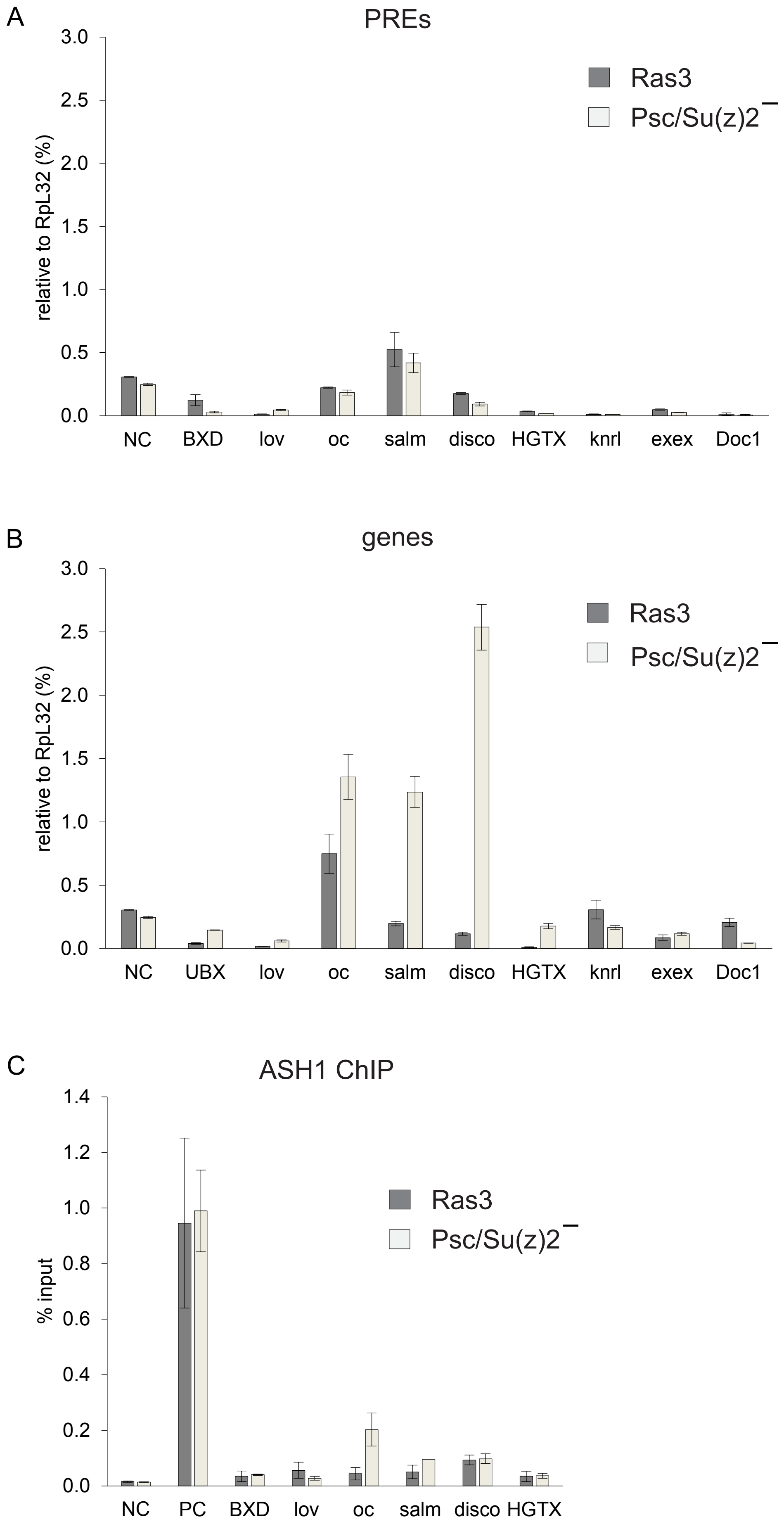
A
PREEs
3.0
2.5
2.0
1.5
1.0
0.5
0.0
relative to RpL32 (%)
Ras3
Psc/Su(z)2⁻
NC
BXD
lov
oc
salm
disco
HGTX
knrl
exex
Doc1
B
genes
3.0
2.5
2.0
1.5
1.0
0.5
0.0
relative to RpL32 (%)
Ras3
Psc/Su(z)2⁻
NC
UBX
lov
oc
salm
disco
HGTX
knrl
exex
Doc1
C
ASH1 ChIP
1.4
1.2
1.0
0.8
0.6
0.4
0.2
0.0
% input
Ras3
Psc/Su(z)2⁻
NC
PC
BXD
lov
oc
salm
disco
HGTX

Figure S4

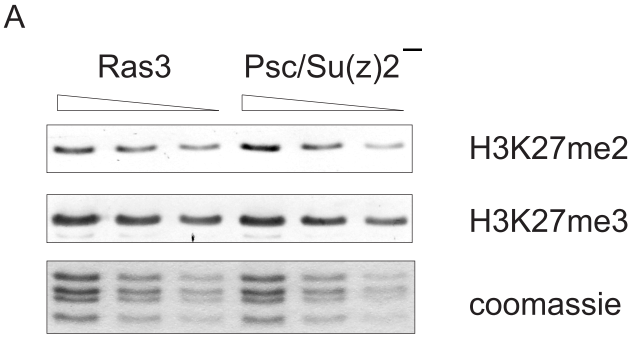
A
Ras3
Psc/Su(z)2⁻
H3K27me2
H3K27me3
coomassie

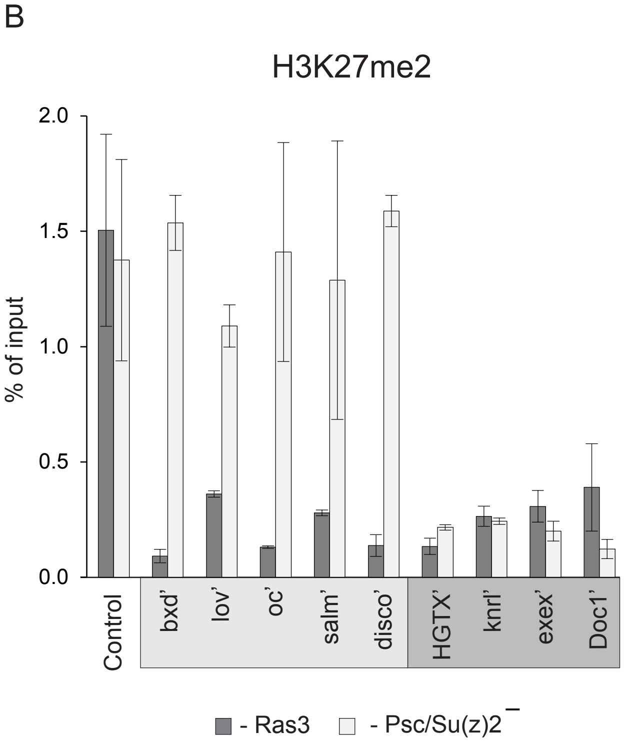
B
H3K27me2
% of input
2.0
1.5
1.0
0.5
0.0
Control
bxd'
lov'
oc'
salm'
disco'
HGTX'
knrl'
exex'
Doc1'
- Ras3
- Psc/Su(z)2⁻

Figure S5

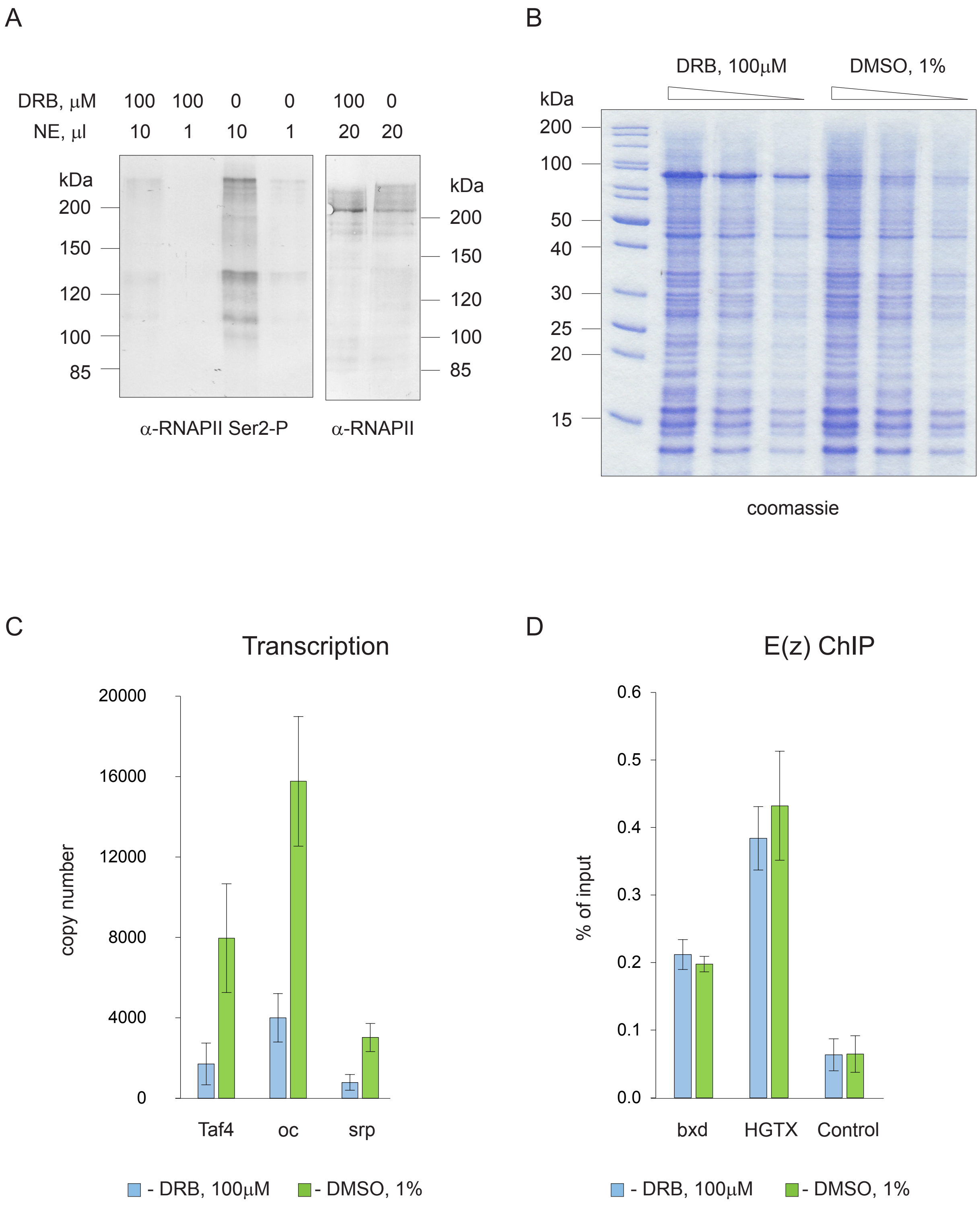

A
DRB, μM 100 100 0 0 100 0
NE, μl 10 1 10 1 20 20
kDa
200
150
120
100
85
α-RNAPII Ser2-P
α-RNAPII
B
DRB, 100μM
DMSO, 1%
kDa
200
100
50
40
30
25
20
15
coomassie
C
Transcription
copy number
20000
16000
12000
8000
4000
0
Taf4
oc
srp
- DRB, 100μM
- DMSO, 1%
D
E(z) ChIP
% of input
0.6
0.5
0.4
0.3
0.2
0.1
0.0
bxd
HGTX
Control
- DRB, 100μM
- DMSO, 1%

Figure S6

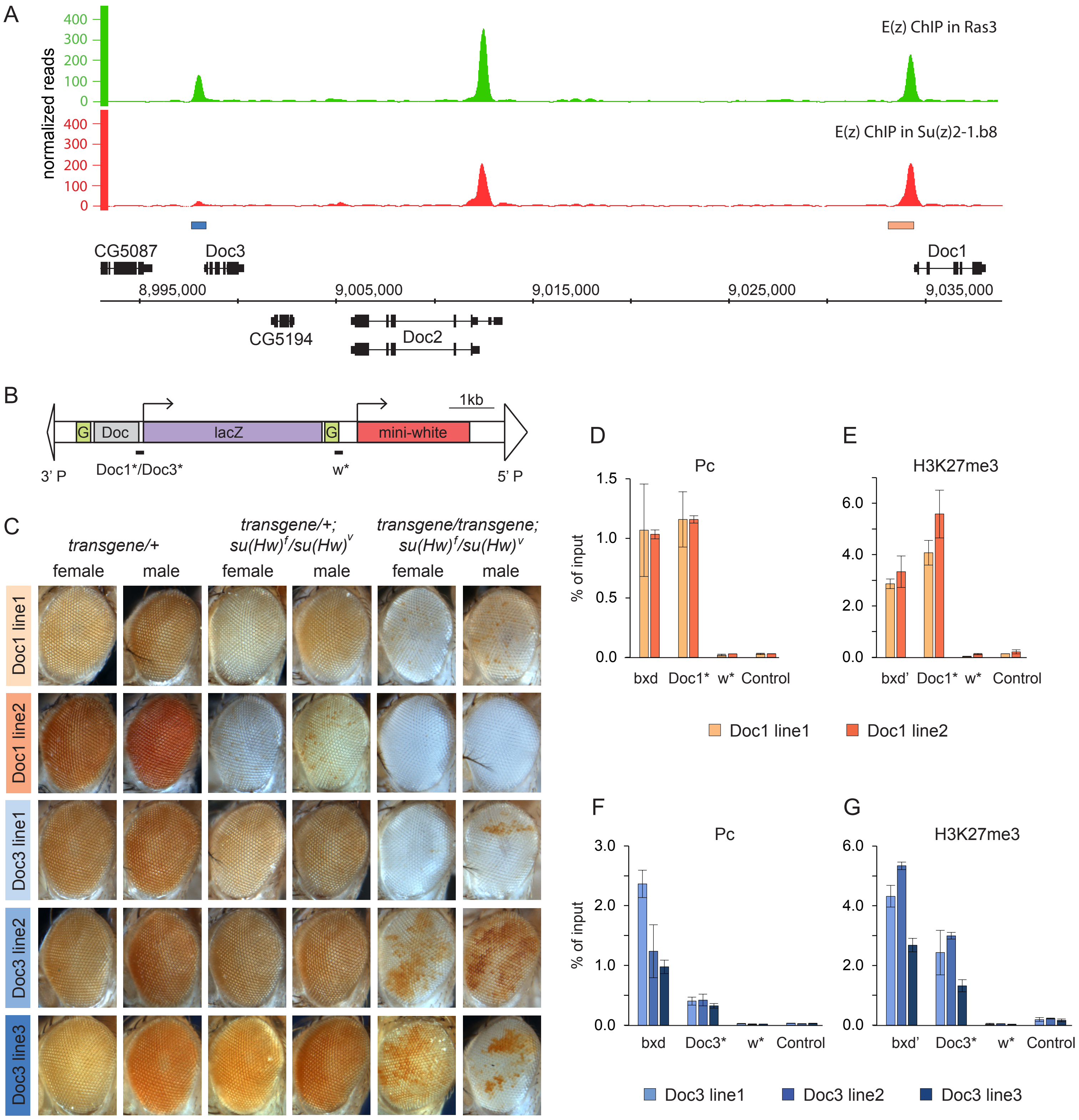

A
normalized reads
400
300
200
100
0
E(z) ChIP in Ras3
E(z) ChIP in Su(z)2-1.b8
CG5087
Doc3
Doc1
8,995,000
9,005,000
9,015,000
9,025,000
9,035,000
CG5194
Doc2
B
G
Doc
lacZ
mini-white
1kb
3' P
Doc1*/Doc3*
w*
5' P
C
transgene/+
transgene/+; su(Hw)f/su(Hw)v
transgene/transgene; su(Hw)f/su(Hw)v
female
male
Doc1 line1
Doc1 line2
Doc3 line1
Doc3 line2
Doc3 line3
D
Pc
% of input
bxd
Doc1*
w*
Control
E
H3K27me3
bxd'
Doc1 line1
Doc1 line2
F
G
Doc3*
Doc3 line1
Doc3 line2
Doc3 line3

Figure S7

A

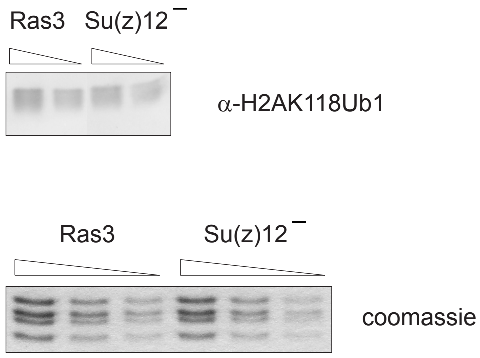

Ras3
Su(z)12⁻
α-H2AK118Ub1
Ras3
Su(z)12⁻
coomassie

B

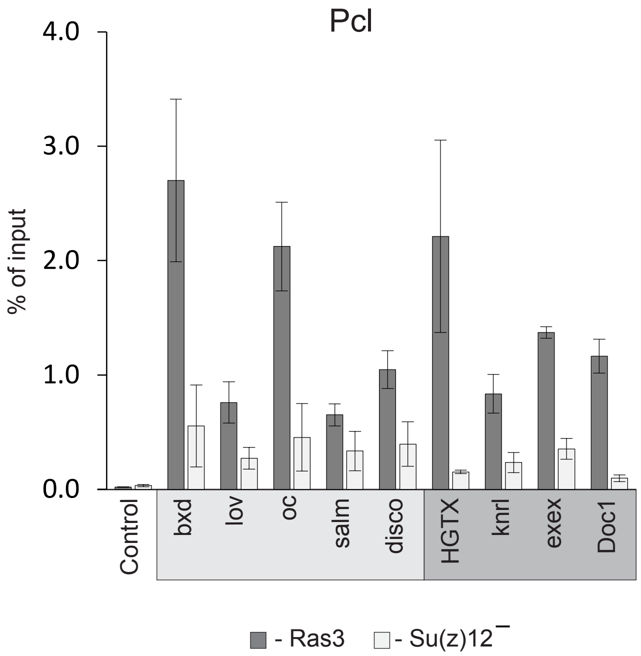

Pcl
4.0
3.0
2.0
1.0
0.0
% of input
Control
bxd
lov
oc
salm
disco
HGTX
knrl
exex
Doc1
- Ras3
- Su(z)12⁻

Figure S8

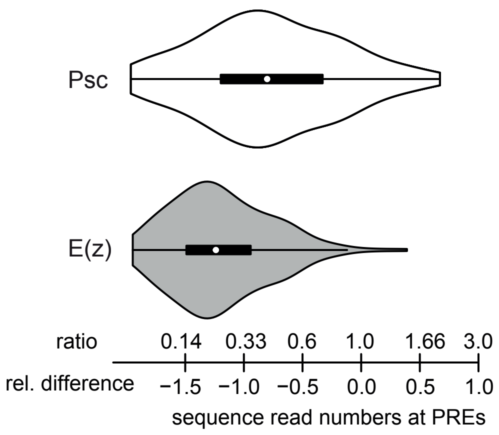
Psc
E(z)
ratio
0.14
0.33
0.6
1.0
1.66
3.0
rel. difference
−1.5
−1.0
−0.5
0.0
0.5
1.0
sequence read numbers at PREs

Figure S9

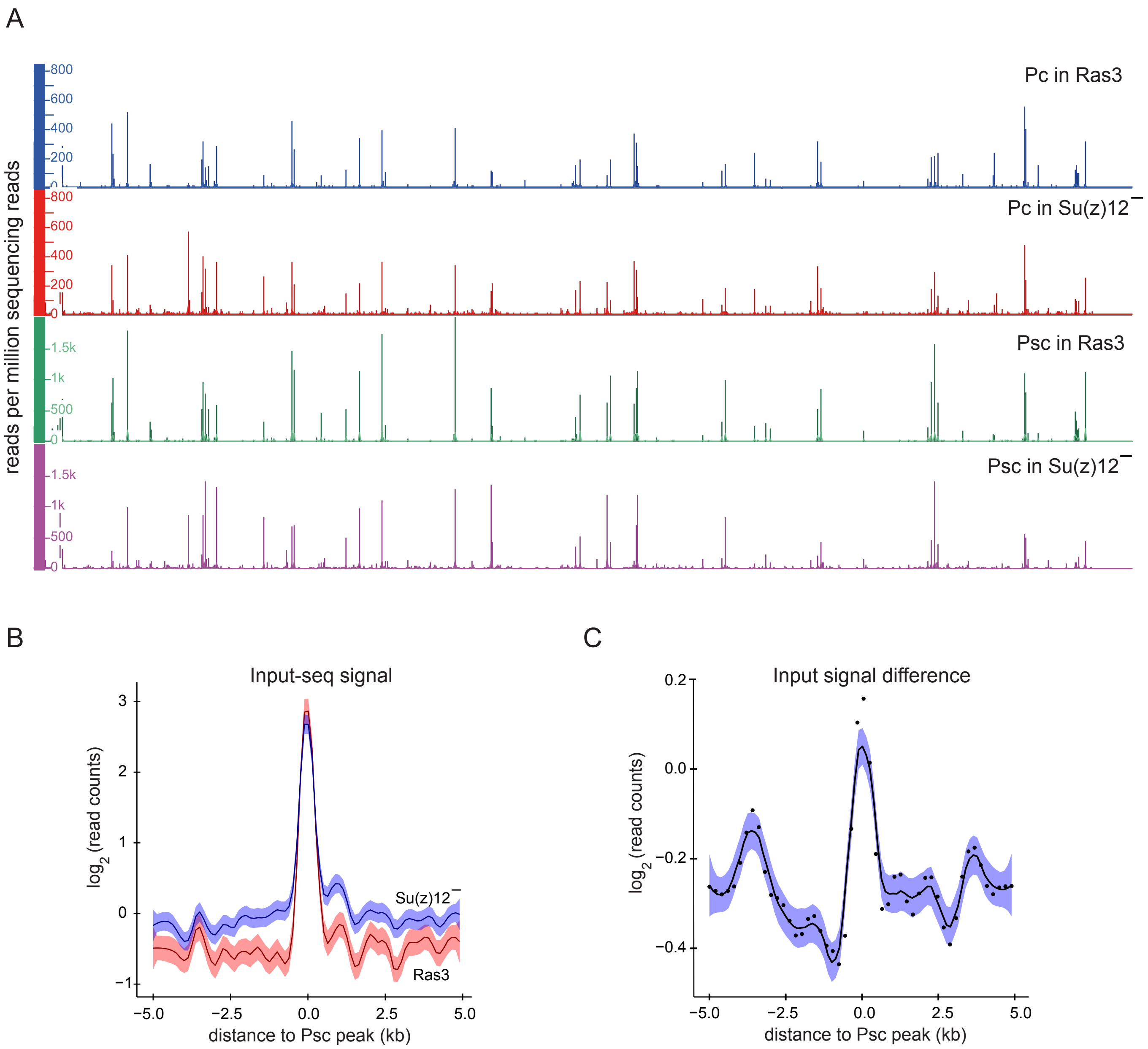
A
reads per million sequencing reads
Pc in Ras3
Pc in Su(z)12⁻
Psc in Ras3
Psc in Su(z)12⁻
800
600
400
200
0
1.5k
1k
500
B
Input-seq signal
log2 (read counts)
Su(z)12⁻
Ras3
distance to Psc peak (kb)
C
Input signal difference
log2 (read counts)
distance to Psc peak (kb)

Table S1. The list of oligonucleotide primers

Amplicon name	Primer name	Primer sequence, 5'-3'	T annealing	Product size (bp)
RNAi				
	dRingRi1.1	**CTAATACGACTCACTATAGGGAG**ACACAGTTCTCCTTGAGTGC	55	
**RING1-Ri**	dRingRi1.2	**CTAATACGACTCACTATAGGGAG**ATGCTGAAGAAGACGATGAC	55	951
	l(3)73Ah-Ri1.1	**CTAATACGACTCACTATAGGGAG**CATAACGTGCAAGATCTGCGG	55	
**L(3)73Ah-Ri**	l(3)73Ah-Ri1.2	**CTAATACGACTCACTATAGGGAG**AGACGAACTTCAGCGTGTGA	55	817
	calypso-Ri1.1	**CTAATACGACTCACTATAGGGAG**CGACTTGCCATTCTTAGCGG	55	
**Calypso-Ri**	calypso-Ri1.2	**CTAATACGACTCACTATAGGGAG**AGAACAAGGGGCTGGCTATT	55	857
	LZRi1.1	**CTAATACGACTCACTATAGGGAG**GGCCACCGATATTATTTGCC	55	
**LZRi**	LZRi1.2	**CTAATACGACTCACTATAGGGAG**CACACTGAGGTTTTCCGC	55	703
RT-qPCR				
	RpL32-ex1.1	TGGGCGATCTCGCCGCAGTA	55	
**Control**	RpL32-ex1.2	CAGAGTGCGTCGCCGCTTCA	55	174
	Pc-ex2.1	GCCGTAACTAAAACTAGCCA	50	
**Pc**	Pc-ex2.2	CGAGCCCATAGACTCATTAC	50	178
	dRingEx1.1	CATTTTCACACCAATTCTGA	55	
**RING1**	dRingEx1.2	CGGTCACACTTCAAGCTAAT	55	95
	EZ-ex1.1	GATCGCCGGTGACCATCATA	55	
**E(z)**	EZ-ex1.2	GTTGTGGATGCCACTCGGAA	55	97
	PSCEX1.1	TCCATTGACCATTCGCACAG	55	
**Psc**	PSCEX1.2	TTTCACCTTGATGGGTTTCAG	55	142
	l(3)73Ah-ex4.1	GGGTCGAGCTCTAACCACTGT	55	
**l(3)73Ah**	l(3)73Ah-ex4.2	GGTTTCGAGGGGACTAACACT	55	121
	Calypso-ex2.1	CTCATGCCTTTGGTGTGGGT	55	
**Calypso**	Calypso-ex2.2	CCAGCATTTTCTTCGCCCAG	55	142
	U-ex1.1	GTCTTTGTAGCCATTCACCG	55	
**UBX**	U-ex1.2	CCCTATGCCAACCACCATC	55	155
	Tkr-ex1.1	CCAACCCCGGAAATCCTTGA	63	
**lov**	Tkr-ex1.2	ATCCGGGCTCACAAAATGGT	63	111
	oc-ex1.1	GAGAAGGCCTGCGCGGACAC	55	
**oc**	oc-ex1.2	TCGGGCTACACGGCCTCCAA	55	77
	salm-ex1.1	GCTGCGAGCGCGATCCTTCA	55	
**salm**	salm-ex1.3	ACGTTCTGTGTGCGATGCGT	55	120
	disco-ex1.1	CTGAACACCATATTGCAGC	55	
**disco**	disco-ex1.2	GTCAGTTTATCAATCCAGCC	55	157
	HGTX-ex1.1	CACCGACTCCTTGACCCTTC	55	
**HGTX**	HGTX-ex1.2	CTGAGTTTTCTCGGTCCCCC	55	73
	knrl-ex1.1	TAATTCCATCCGCTCTCGCC	55	
**knrl**	knrl-ex1.2	AACTACCGCCACACTCCAAG	55	129
	exex-ex1.1	GGCCTTTACATCGCAGCAAC	55	
**exex**	exex-ex1.2	CGTTTGGGCCGACTCAGATA	55	75
	Doc1-ex1.1	TGAGCTATGGACCCAGTGGA	55	
**Doc1**	Doc1-ex1.2	CGAGGCCACAGGACTAACAG	55	140
**Taf4 (nascent**	Taf4-ex-nt1.1	GTTCCGTTTTGGTTCGTCACA	55	
**transcript)**	Taf4-ex-nt1.1	AGACAGTTACACAACGCAGCA	55	109
**oc (nascent**	oc-His-1.1	TCCCGCTCCCCCAAAAACTACT	55	
**transcript)**	oc-His-1.2	CCAGCAAGGAATGTGGAGCCGA	55	82

**srp (nascent transcript)**	srp-1.1	AGCACCGACTTCCCCACC	55	
	srp-1.2	CAATCAAATGCGGGTCGCG	55	204
ChIP-qPCR				
**Control**	3L-nc-1.1	AAACTGCCGCACGACGGAGG	55	
	3L-nc-1.2	CGGCCCTGCAACGCCTGTAA	55	82
**bxd**	BP-3	GCCATAACGGCAGAACCAAAG	55	
	BP-4	ATGAGGCCATCTCAGTCGC	55	258
**bxd'**	FM8a.1	GGAGTAGCGTGCTACGGGGG	55	
	FM8a.2	CGACATGAAATGTTGCACGGCCA	55	81
**lov**	Tkr-1.1	CGAACGCTAGGCAGAAAGA	55	
	Tkr-1.2	CCAACTCGTGTGCAGCAAAT	55	90
**lov'**	Tkr-His1.1	TGAACAACAGTTCGCCTGGA	55	
	Tkr-His1.2	TGCTTGGTCAAGACTGCGAG	55	70
**oc**	oc-1.1	CTACCGCGCGCCAGAGTGAG	55	
	oc-1.2	AGTCAGGTTGTAAGCGGCGTGC	55	122
**oc'**	oc-His2.1	GGAGGCTAGGTAGGCGTAGA	55	
	oc-His2.2	ATCCCGGTAGCCTTGGTACT	55	102
**salm**	salm-1.1	TCCTTCCGCCTTTTGCGGCTC	55	
	salm-1.2	GGCTCGTTGGCGGGGGAAG	55	120
**salm'**	salm-His2.1	TTTGGAGACGTGCCCACTTT	55	
	salm-His2.2	CAACACTCCCCCGATTCCAA	55	84
**disco**	disco-1.1	CGCTGCTGCGACGTCACTTT	55	
	disco-1.2	TCGTGCGGGTGCGATAGGGA	55	138
**disco'**	disco-His-1.1	GTGCAGCAACCTTTATGCTGCCT	55	
	disco-His-1.2	GGCGGCGCCGAATTGAAAAGT	55	150
**HGTX**	HGTX-1.1	CATGACGTGTTGGCATGTCG	55	
	HGTX-1.2	CACAGAGCGGCTGTAAGTGA	55	103
**HGTX'**	HGTX-His1.1	ATTTAGTGGCCCCCATGTCC	55	
	HGTX-His1.2	ATGTCCGTGGAGTGGAGAGA	55	72
**knrl**	knrl-1.1	ATCGTATTAGCGGCTTGCCA	55	
	knrl-1.2	GCTGGAAGTGGGCAATGTTC	55	111
**knrl'**	knrl-His1.1	TCGCCCCTTTCTGGTTACAC	55	
	knrl-His1.2	CCGATTCCGCATTCTGCAAG	55	122
**exex**	exex-1.1	TCTGTCGCGCTTTCGTGTTA	63	
	exex-1.2	GGTGCAATCAGTACCGCTCT	63	94
**exex'**	exex-His1.1	CAACGATGACCAAATCGCCC	55	
	exex-His1.2	TGGTCACCGGAAATGGGATG	55	102
**Doc1**	Doc1-1.1	CTCCGCTGCTCACAAAATGG	55	
	Doc1-1.2	GCAGCTACGCTTGGAAAACC	55	146
**Doc1'**	Doc1-His1.1	CGAGGGACTTGGATCGTCTG	66	
	Doc1-His1.2	ACTGAGAAGGACGCGACAAG	66	86
**FM2**	FM2.1	TGCTTCTGTTCATTATTGTCTC	55	
	FM2.2	GAGTTCCGTGATTGGATTGC	55	213
**FM3**	FM3.1	GTTTTTCAATATGCCGATTAGC	55	
	FM3.2	AATATAATGTATCTATGAGTCAG	55	227
**FM4**	FM4.1	AGCAATTTGTCACCGCAAGG	55	
	FM4.2	GGATTTTGAGTGCGTTCTTCC	55	216
**BP**	BP-3	GCCATAACGGCAGAACCAAAG	55	
	BP-4	ATGAGGCCATCTCAGTCGC	55	258
	FM5.1	TCTCTCGCTGGTTGTGTTTG	55	

**FM5**	FM5.2	CGTGCGGTATGGAGAGATG	55	189
	FM7.1	GGGTCAAAGAACTCGAAATATG	55	
**FM7**	FM7.2	ATTTCAATGTATTCGGTTATTAAG	55	245
	FM8a.1	GGAGTAGCGTGCTACGGGGG	55	
**FM8**	FM8a.2	CGACATGAAATGTTGCACGGCCA	55	81
	Doc1-2.1	GAGTTAGCACTCGAGCGGAG	55	
**Doc1***	pH-Pelican-REV1	ATTTATACTCCGGCGCTCCT	55	94
	Doc3-1.1	GTGTTCCAGACGTCGATTGC	55	
**Doc3***	Pelican-REV1	TGCTTTAGCAGGCTCTTTCG	55	112
	Pelican-FWD1	TGCCATACCATTTAGCCGATCA	55	
**w***	Pelican-REV2	AGTTCAATGATGTCCAGTGCAG	55	125
	attB_BXD-PRE-1-[illegible]	CAAGCGGGCAGCATAAAAC	55	
**BXD***	w-prom1	CTGCACTGGATATCATTGAAC	55	161
	HGTX-2.1	CTCAGTCGCATGATATCCCT	55	
**HGTX***	w-prom1	CTGCACTGGATATCATTGAAC	55	288
	knrl-2.1	TCGACCGTATGGTAATCGCT	55	
**knrl***	w-prom1	CTGCACTGGATATCATTGAAC	55	276
	3Lnc-1.3	GATGGAGCGACACGATAGCA	55	
**control***	w-prom1	CTGCACTGGATATCATTGAAC	55	160
	pnt-1.1	GCCCAGTCCCAGCGAAGCAG	55	
**pnt**	pnt-1.2	GCAGGGATTCGCCAACGGGA	55	85

Table S2. The list of antibodies

Antigene	Epitope, aa / clone name	Type of antibody	Reference/Company/Catalog#	Western dilution	Amount used for ChIP
E(z)	8-190	rabbit polyclonal	Kahn et al., 2006	1:1000	3ug
Pcl	191–354	rabbit polyclonal	Poux et al., 2001	1:1000	3ug
Psc	819-926	rabbit polyclonal	Poux et al., 2001	1:2000	2ug
Pc	191–354	rabbit polyclonal	Poux et al., 2001	1:2000	2ug
Pcl	113–537	rabbit polyclonal	O´Connell et al., 2001	1:2000	5ug
dRING_Q3200		rabbit polyclonal	modENCODE Consortium et al., 2010	1:5000	5ug
dSfmbt_Q2642	14-113	rabbit polyclonal	modENCODE Consortium et al., 2010	1:3000	
Pho	93-276	rabbit polyclonal	Poux et al., 2001		50ul
Ash1	1756-1855	rabbit polyclonal	this study, validated by ChIP in Ash1 deficient cultured cells*		5ug
H3K27me3		mouse monoclonal	Abcam, #ab6002, lot#GR77445-3, 1ug/ul		0.5ug
H3K27me3		rabbit polyclonal	Upstate, #07-449	1:4000	
Di-methyl-Histone H3 (K27)		rabbit moloclonal	Cell Signaling, #9728S, Lot#4	1:3000	3ul
H3K27me1		rabbit polyclonal	Diagenode, #pAb-045-050	1:1000	
Ubiquityl-Histone H2A (Lys119)	D27C4	rabbit moloclonal	Cell Signaling, #8240	1:5000	5ug
H3		rabbit polyclonal	Abcam, #ab1791	1:100000	
Anti-RNA polymerase II CTD repeat YSPTSPS antibody	8WG16	mouse monoclonal	Abcam, ab817	1:500	
Phospho-Rpb1 CTD (Ser2)	E1Z3G	rabbit moloclonal	Cell Signaling, #13499	1:5000	

* validation of anti-Ash1 antibodies, 3L-nc1 - intergenic region, BXD-PRE - PRE in repressed state, pnt - PcG target gene in active state

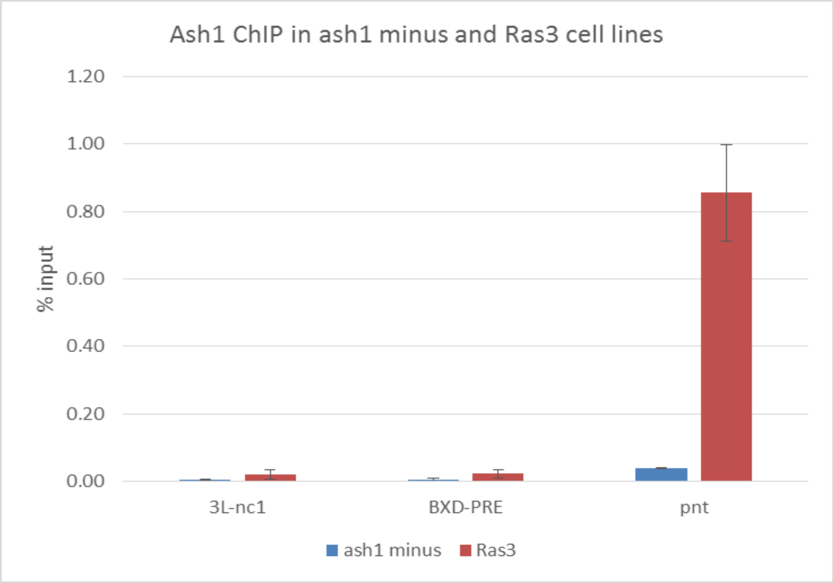

Table S3. Relative differences in E(z) ChIP-seq signals at PREs between Su(z)2-1.b8 and Ras3 cells

PRE position*			Relative difference in sequencing reads between Su(z)2-1.b8 and Ras3 cells (average for two biological replicates)		corrsponding PcG target gene
chromosome	start	stop	for E(z) ChIP samples	for Input samples	
chr2L	15111038	15112038	0.39023719	-0.01351885	CG15269
chr2R	20229351	20230351	0.097082462	-0.034390109	bs
chr2L	5290	6290	-0.048085475	-0.218056837	CG11023
chr3L	9033540	9034540	-0.146171061	-0.192901096	Doc1
chr3R	12239121	12240121	-0.24052466	0.043769344	ss
chr3L	7940403	7941403	-0.240958253	-0.14627894	exex
chr3L	20614587	20615587	-0.308328587	-0.304758214	knrl
chr3R	20920186	20921186	-0.342738266	0.10997004	fd96cb
chr2R	13709561	13710561	-0.445441055	-0.126968917	grh
chr3R	17204463	17205463	-0.458656895	-0.312949116	tin
chr2R	18129478	18130478	-0.507332065	0.095605387	dve
chr3R	678193	679193	-0.532301721	-0.302185899	opa
chr2L	18881109	18882109	-0.563348014	0.075588434	tup
chr3L	9011622	9012622	-0.584378297	-0.494220062	Doc2
chr3R	12248773	12249773	-0.590498191	-0.239186458	ss
chr2L	1972530	1973530	-0.599212497	-0.167568879	CG31670
chr3R	17386852	17387852	-0.624059626	-0.274435573	slou
chr3L	14170673	14171673	-0.638136678	0.034043334	D
chr3R	7179390	7180390	-0.650022226	-0.556394677	Pros
chrX	4305183	4306183	-0.651350319	-0.164353972	bi
chr2R	10686482	10687482	-0.683458384	-0.332889313	kn
chr3L	12572833	12573833	-0.704964319	-0.332643806	ara
chr3R	9726877	9727877	-0.720880465	-0.396605191	ems
chr2R	5082022	5083022	-0.722936592	0.259843872	unpg
chr2L	3606249	3607249	-0.729891722	-0.063920017	odd
chr3L	12427253	12428253	-0.733112715	0.08563378	toe
chr3L	19656435	19657435	-0.741318843	-0.053955474	tey
chr3L	14571686	14572686	-0.779625249	0.251986833	HGTX
chr3R	2567414	2568414	-0.779969275	-0.385529821	pb
chr2R	20701204	20702204	-0.791195045	-0.217486849	Dll
chr3R	8889403	8890403	-0.796388378	-0.228593737	sim
chr3L	3845599	3846599	-0.890469205	-0.405701341	Awh
chr3R	20906136	20907136	-0.926565344	0.088469468	fd96Ca
chr3R	17324852	17325852	-0.931964925	-0.273970898	C15
chr2R	21002787	21003787	-0.940912399	-0.132848968	Tkr
chr3R	12229023	12230023	-0.953218765	-0.097504979	ss
chr2L	3824916	3825916	-0.959350661	-0.040952295	slp1
chr2L	6820973	6821973	-0.959556678	0.021715579	sens-2
chr3R	660532	661532	-0.966717636	-0.33288126	opa

chr3R	9700307	9701307	-0.970480185	-0.340556415	E5
chr2L	3835576	3836576	-0.976351823	0.064117961	slp2
chr3L	6896535	6897535	-0.987929551	-0.297915519	Prat2
chr3L	14592362	14593362	-0.988768668	-0.49702735	HGTX
chr3R	6442098	6443098	-1.008346711	-0.441904109	hth
chr3L	14124366	14125366	-1.026474249	-0.275327043	Sox21b
chr3L	12602148	12603148	-1.037450207	-0.346457885	caup
chr2R	20948836	20949836	-1.06871344	0.140107742	gsb
chr3R	13382892	13383892	-1.100189853	-0.149261103	Hmx
chrX	8518322	8519322	-1.104908761	0.176867531	oc
chr2R	3305913	3306913	-1.138185193	-0.819984423	so
chr2R	10345135	10346135	-1.150461159	-0.071867133	Oaz
chrX	5895108	5896108	-1.170667762	0.393398124	CG4766
chr3R	11851364	11852364	-1.173897328	-0.239905627	pnr
chr3R	17259534	17260534	-1.17425819	0.134247764	lbl
chr3R	4016246	4017246	-1.175809259	0.130412603	grn
chr2L	16485973	16486973	-1.188214235	-0.344115376	dac
chr3R	2503774	2504774	-1.194994244	-0.297639164	lab
chr3L	8997729	8998729	-1.199786184	-0.686166533	Doc3
chr3L	12460807	12461807	-1.202986735	-0.072774107	eyg
chr2L	3662269	3663269	-1.203261154	-0.116965128	CG34340
chr3R	8106072	8107072	-1.208119217	-0.17141146	svp
chr2L	18795571	18796571	-1.222544203	-0.315577151	ham
chr3R	4006950	4007950	-1.226011975	-0.477552043	grn
chr2R	16790601	16791601	-1.230276343	-0.688230304	otp
chrX	5907251	5908251	-1.233912713	0.26462187	mab-21
chr3R	25516149	25517149	-1.235126263	-0.065657068	dmrt99B
chr3L	12645981	12646981	-1.240544024	-0.051058026	CR43431
chr2L	12617692	12618692	-1.250687491	-0.253112832	nub
chr3R	12589291	12590291	-1.254781686	0.110758504	Ubx (bxd-PRE)
chr3L	21472597	21473597	-1.271919049	0.084566739	croc
chr2R	21102468	21103468	-1.279815745	-0.077983774	Kr
chrX	8700536	8701536	-1.280496984	0.279502238	Lim1
chr4	528607	529607	-1.286536632	0.097417561	zfh2
chr3R	24372594	24373594	-1.292441226	-0.067522851	fkh
chr2R	8771483	8772483	-1.29991978	-0.228004619	vg
chr2L	5460902	5461902	-1.302647643	0.052586652	mid
chr2L	5403363	5404363	-1.304611626	-0.276189883	H15
chr3L	12685254	12686254	-1.322299872	-0.102282151	mirr
chr2R	20939693	20940693	-1.324643766	-0.296817409	gsb-n
chr3R	2673473	2674473	-1.338002069	-0.205305131	Scr
chr3R	6490434	6491434	-1.343719028	-0.607683888	CG34304
chr2L	12586427	12587427	-1.345972545	0.039139529	nub
chr2L	7306432	7307432	-1.351508645	-0.033981526	wg
chr2L	8825422	8826422	-1.356806915	-0.134081623	SoxN
chr2L	6833299	6834299	-1.364783832	-0.037343626	sens-2
chr3R	17271782	17272782	-1.366917594	-0.221355789	lbe

chrX	16041559	16042559	-1.369688863	-0.548175747	disco-r
chr3R	2718698	2719698	-1.375561182	-0.320027889	Antp
chr2L	5421429	5422429	-1.380129374	-0.336810952	H15
chr3R	26737738	26738738	-1.382238539	-0.166033215	Ptx1
chr3R	2617004	2618004	-1.387264582	0.113318701	Dfd
chr2R	8791327	8792327	-1.391011281	-0.503132145	vg
chrX	17683194	17684194	-1.408436524	-0.378590855	OdsH
chr2L	16422343	16423343	-1.408947542	-0.320372259	snoRNA:Me28S-A2958
chr2R	1613509	1614509	-1.421110672	-0.510984985	ap
chr2R	7415510	7416510	-1.42328651	-0.371472843	en
chrX	8544485	8545485	-1.4339559	-0.094554267	oc
chr2L	3538793	3539793	-1.435333107	0.088275278	drm
chr2R	15155951	15156951	-1.436296383	0.070163752	rib
chr3R	25381426	25382426	-1.443296348	-0.175228689	Dr
chrX	5454405	5455405	-1.449234258	0.078224444	Vsx2
chr2L	594635	595635	-1.449634413	0.055208702	Gsc
chr3R	12683393	12684393	-1.453591011	0.199286604	Abd-B (iab5-PRE)
chrX	5487778	5488778	-1.462040072	-0.131109254	Vsx1
chrX	16111356	16112356	-1.466784732	-0.411340307	disco
chr2R	7362305	7363305	-1.470632037	-0.36987238	inv
chrX	9587591	9588591	-1.489107614	-0.006431672	btd
chr3R	12795584	12796584	-1.490959802	0.148710004	Abd-B (iab9-PRE?)
chr3L	1177848	1178848	-1.493803855	-0.604312284	bab2
chr2L	20770009	20771009	-1.501890395	0.103488448	cad
chr2R	3918315	3919315	-1.520977207	-0.21760822	Optix
chr2L	11447125	11448125	-1.542027808	-0.20517641	salm
chr3L	6981574	6982574	-1.562343182	-0.430092093	bin
chr3R	4158984	4159984	-1.59070818	-0.310213444	Poxm
chr2R	3905947	3906947	-1.59727804	-0.179511956	Optix
chr2R	7356552	7357552	-1.604264923	-0.191580119	inv
chr3R	2788820	2789820	-1.60541527	-0.045021655	Antp
chr2L	20782896	20783896	-1.625062679	0.323662745	cad
chr3R	12528230	12529230	-1.638650324	0.143139099	Ubx (bx-PRE)
chrX	17662071	17663071	-1.642867006	-0.617593942	unc-4
chr3R	2738487	2739487	-1.644558646	-0.3350898	Antp
chr3L	377833	378833	-1.653789164	-0.54880123	trh
chr3R	2826134	2827134	-1.654004762	-0.331055168	Antp
chr3R	12672528	12673528	-1.677201837	0.156035662	abd-A (iab4-PRE)
chr2R	11558873	11559873	-1.691425089	-0.481582105	fus
chr3R	12655438	12656438	-1.715390317	-0.222313155	abd-A (iab3 PRE)
chr3R	2862935	2863935	-1.721605897	-0.50560921	Antp
chr3R	12707691	12708691	-1.741765471	-0.238736092	Abd-B (Fab6-PRE)
chr3R	12695158	12696158	-1.753221504	0.007045643	Abd-B (Mcp-PRE)
chr3R	6447771	6448771	-1.809855281	-0.880848309	hth
chr2R	10678120	10679120	-1.825251803	-0.533463981	kn
chr4	724688	725688	-1.836627001	-0.936932414	ey
chr3R	12637297	12638297	-1.854100047	-0.326804773	abd-A (iab2-PRE)

chr3R	2755700	2756700	-1.868966644	-0.423310675	Antp
chr2L	1643905	1644905	-1.893941094	-0.032727707	CG31666

* The coordinates are in Dm3, 2006 genome release

Table S4. Inverse-PCR mapping of Doc transgenes

Fly line	Chromosome arm	Coordinates (2006)
Doc1 line 1 (DocF3s1-1B)	2L	9437447
Doc1 line 2 (DocF3s1-4)	2R	2544882
Doc3 line 1 (DocF11s1-3)	2R	13340141
Doc3 line 2 (DocF11s1-9)	2R	repeated sequence
Doc3 line 3 (DocF11s1-10)	2L	5986350

Table S5. Relative differences in E(z) ChIP-seq signals between Su(z)2-1.b8 and Ras3 cells at PRE defined from Ras binding profiles.

PRE position*			Relative difference in sequencing reads between Su(z)2-1.b8 and Ras3 cells (average for two biological replicates)	corrsponding PcG target gene
chromosome	start	stop	for E(z) ChIP samples	
chr2L	15111370	15111629	0.39023719	CG15269
chr2R	20230030	20230389	0.097082462	bs
chr3L	9034052	9034592	-0.14617106	Doc1
chr3R	12239531	12239850	-0.24052466	ss
chr3L	7940956	7941380	-0.240958254	exex
chr3L	19675986	19676442	-0.286802832	CG8765
chr3L	20614693	20615295	-0.308328587	knrl
chr3R	20920230	20920721	-0.342738266	fd96cb
chr2R	13709652	13710298	-0.445441055	grh
chr3R	17204398	17204885	-0.458656895	tin
chr3R	9702800	9703291	-0.502979906	E5
chr2R	18129365	18131598	-0.506306242	dve
chr3R	677857	678677	-0.530265844	opa
chr2L	18881127	18881948	-0.563348014	tup
chrX	9623038	9623675	-0.569935632	Sp1
chr3L	6782832	6783343	-0.573247034	vvl
chr3L	9012224	9012809	-0.584378297	Doc2
chr3R	12248987	12249461	-0.590498191	ss
chr2L	1972535	1973141	-0.599212497	CG31670
chrX	18206421	18207101	-0.605567312	CG6023
chr3R	17387277	17387727	-0.624059626	slou
chr3R	7197778	7198569	-0.632572911	pros
chr3R	8070262	8070471	-0.632583506	Cyp313a3
chr3L	14170572	14171296	-0.638136678	D
chr2R	19508883	19509297	-0.641957867	CG4019
chr3R	7179101	7179685	-0.650022226	Pros
chrX	4305279	4305881	-0.651350319	bi
chr2R	10686390	10687325	-0.683458384	kn
chrX	2030869	2031272	-0.687180361	ph-p
chr2R	19519753	19520230	-0.688653237	retn
chr3L	12573211	12573895	-0.704964319	ara
chr3R	9727142	9727684	-0.720880465	ems
chr2R	5082303	5082822	-0.722936592	unpg
chr2L	3606489	3607094	-0.729891722	odd
chr3L	19656602	19657174	-0.741318843	tey
chr3L	11002127	11002699	-0.776183243	klu
chr3L	14571975	14572488	-0.779625249	HGTX
chr3R	2567233	2568185	-0.779969275	pb
chr2R	20701940	20702501	-0.788538538	Dll
chr2L	9581579	9582057	-0.788917099	gcm
chr2L	1970362	1970880	-0.795800226	Der-1
chr3R	8889743	8890347	-0.796388378	sim
chr3L	12426860	12427916	-0.815657021	toe

chr3R	24410963	24411495	-0.826656728	fkh
chr2L	6536052	6536460	-0.878202894	eya
chr3R	26734766	26735163	-0.885478507	Ptx1
chr3L	3845713	3846205	-0.890469205	Awh
chr2L	3836502	3836947	-0.913281728	slp2
chr3R	8104718	8105185	-0.915624086	svp
chr3R	20906095	20906581	-0.926565344	fd96Ca
chr2R	7590518	7590890	-0.928510088	Ir48b
chr3R	17325073	17325636	-0.931964925	C15
chr3R	13947195	13947817	-0.939832875	sr
chr3R	11818785	11819216	-0.944052635	srp
chr2R	5875793	5876217	-0.948872185	TER94
chr2R	21002813	21003424	-0.949125027	Tkr
chr3R	12229202	12229872	-0.953218765	ss
chr2L	3825334	3825786	-0.959350661	slp1
chr2L	6821575	6822111	-0.959556678	sens-2
chr3R	660947	661454	-0.966717636	CG14659
chr3R	9700578	9701217	-0.970480185	E5
chr3L	6896977	6897674	-0.987929551	Prat2
chr3L	14592606	14592965	-0.988768668	HGTX
chrX	4318933	4319612	-0.996803629	bi
chr3R	6442415	6443105	-1.008346711	hth
chr4	865042	865466	-1.014007184	Sox102F
chr2L	21828179	21829074	-1.016675409	tsh
chr3L	12683858	12684198	-1.021112415	mirr
chr3L	14124711	14125162	-1.026474249	Sox21b
chr2L	3835245	3835644	-1.031606053	slp2
chr3L	12602287	12602933	-1.037450207	caup
chr2R	20949155	20949539	-1.06871344	gsb
chr2R	4955882	4956261	-1.098051017	ana
chr3R	13383183	13383577	-1.100189853	Hmx
chrX	16042983	16043453	-1.104408524	disco-r
chrX	8518806	8519329	-1.104908761	oc
chr3L	21469508	21469951	-1.108321801	croc
chr3R	17285686	17286153	-1.115292251	lbe
chr2L	18936072	18936508	-1.132586121	ssp3
chrX	16110595	16111958	-1.134840138	disco
chr2R	3305938	3307010	-1.138185193	so
chr2R	10345363	10346045	-1.150461159	Oaz
chrX	4320592	4321328	-1.165838586	bi
chrX	8699672	8701052	-1.166939398	Lim1
chr3R	3135271	3135724	-1.167180226	rn
chrX	5895173	5895579	-1.170667762	CG4766
chr3R	11851519	11851955	-1.173897328	pnr
chr3R	17259805	17260339	-1.17425819	lbl
chr3R	4016498	4017064	-1.175809259	grn
chr3R	2675550	2676079	-1.193688265	Scr
chr3R	2504162	2504614	-1.194994244	lab
chr2L	11358486	11359050	-1.195259699	salr
chr2R	5866450	5866987	-1.196194751	eve

chr3L	8997832	8998243	-1.199786184	Doc3
chr3L	12460998	12461488	-1.202986736	eyg
chr2L	3662365	3662771	-1.203261154	CG34340
chr3R	8106425	8106941	-1.208119217	svp
chr2R	18933556	18933995	-1.219306806	twi
chr2L	18795879	18796691	-1.222544203	ham
chr2L	16485569	16486613	-1.225800689	CG4580
chr3R	4007014	4007779	-1.226011975	grn
chrX	5907070	5907631	-1.226463461	mab-21
chr2R	16790893	16791490	-1.230276343	CG9235
chr3R	12759885	12760543	-1.235296742	Abd-B
chr2L	14490333	14490960	-1.23784642	noc
chr3R	727065	728091	-1.238529284	Gnf1
chr3L	12646172	12646652	-1.240544024	mirr
chrX	8651821	8652367	-1.242655553	Lim1
chr2L	12617771	12618589	-1.250687491	nub
chr4	525767	526194	-1.254772257	zfh2
chr3R	12589541	12590187	-1.254781686	Ubx
chr3L	21472713	21473185	-1.271919049	croc
chr2R	21102665	21103059	-1.279815745	Kr
chr2L	14410293	14410601	-1.284363135	elB
chr4	527867	529481	-1.286536632	zfh2
chr3R	24372820	24373322	-1.292441226	CG12413
chr2R	8771763	8772293	-1.29991978	vg
chr2L	5461114	5461728	-1.302647643	mid
chr2L	22024370	22024863	-1.303100148	tio
chr2L	5403461	5404105	-1.304611626	H15
chr3L	12685787	12686831	-1.322299872	mirr
chr2R	20939757	20940389	-1.324643766	gsb-n
chr3R	2674234	2674740	-1.332825378	Scr
chr2L	3534112	3534582	-1.339629155	Che-13
chr3R	6490660	6491189	-1.343719028	CG6465
chr2L	12085706	12086181	-1.34465627	prd
chr2L	12586850	12587329	-1.345972545	nub
chrX	9586690	9586981	-1.349307856	btd
chr2L	7306724	7307264	-1.351508645	wg
chr2L	8825080	8825767	-1.361789892	SoxN
chr2L	6833488	6834186	-1.364783832	sens-2
chr2L	2448488	2448834	-1.366897304	dpp
chr3R	17272003	17272609	-1.366917594	lbe
chrX	16041494	16042385	-1.369688863	disco-r
chr3R	2718729	2719334	-1.375561182	Antp
chr2L	5421448	5422074	-1.380129374	H15
chr3R	26738009	26738594	-1.382238539	Ptx1
chr3R	2617134	2617676	-1.387264582	Dfd
chr2R	8791707	8792326	-1.391011281	vg
chr2L	3538616	3540427	-1.3956134	drm
chr3L	21577865	21578245	-1.401707326	CG32441
chr2L	12655656	12656153	-1.402615125	pdm2
chr2L	16422631	16423088	-1.408947542	CG5888

chr2R	1614207	1614794	-1.420292367	ap
chr2R	7415312	7416124	-1.42328651	en
chrX	8544621	8545319	-1.4339559	oc
chr2L	11445453	11446449	-1.435275166	salm
chr2R	15156175	15156766	-1.436296383	rib
chr3R	25381690	25382269	-1.443296348	Dr
chr2R	7416925	7417442	-1.44756059	en
chrX	5454368	5455107	-1.449234258	Vsx2
chr2L	594614	595200	-1.449634414	Gsc
chrX	8555081	8555314	-1.454787513	oc
chrX	5487826	5488404	-1.462040072	Vsx1
chr2R	10095712	10096104	-1.464666682	Sox15
chr2R	7362838	7363393	-1.470632037	inv
chrX	17683493	17684642	-1.476072804	OdsH
chrX	9587718	9588331	-1.489107614	btd
chr3R	12795794	12796107	-1.490959802	Abd-B
chr3L	1178008	1178721	-1.493803855	bab2
chr2L	20770305	20770807	-1.501890395	cad
chr2L	21846895	21847955	-1.502817677	tsh
chr2R	19525454	19525805	-1.516203422	retn
chr3R	12785972	12786253	-1.528319113	Abd-B
chr2R	3918206	3918846	-1.531289803	Optix
chr2R	3911853	3912257	-1.535273189	Optix
chr3R	3762459	3762987	-1.548344359	dsx
chr2L	11447479	11447998	-1.552501955	salm
chr3L	21591366	21591957	-1.554870727	AP-2
chr3L	6981892	6982893	-1.562343182	bin
chrX	8510738	8510965	-1.570163102	Caf1-180
chrX	20561468	20561871	-1.590519053	run
chr3R	4159369	4160504	-1.59070818	Poxm
chr2R	3906184	3906830	-1.59727804	CG14762
chr2R	7356866	7357359	-1.604264923	E(Pc)
chr3R	19026683	19027209	-1.615101071	cnc
chr3R	3788356	3789630	-1.621508636	dsx
chr2L	20783064	20783522	-1.625062679	cad
chr3R	12528467	12529058	-1.638650324	Ubx
chrX	17661972	17662715	-1.642867006	unc-4
chr3R	2738495	2739016	-1.644558646	Antp
chr3L	377257	378559	-1.653789164	trh
chr3R	2826238	2827042	-1.654004762	Antp
chr2L	7358858	7359156	-1.656310895	Wnt6
chr3R	12712222	12712538	-1.669939736	Abd-B
chr3R	12672842	12673391	-1.677201837	iab-4
chrX	5473621	5473839	-1.683410509	Vsx1
chr3R	12746086	12746508	-1.689063127	Abd-B
chr2R	11559081	11559392	-1.691425089	fus
chr3R	12655674	12656150	-1.715390317	Abd-A
chr3R	2862836	2863512	-1.721605897	CG1979
chr3R	12725360	12725896	-1.729197434	Abd-B
chr2R	20732353	20732594	-1.737368487	CG3650

chr3R	12708034	12708699	-1.741765471	Abd-B
chr2R	15141546	15141993	-1.750744739	CG33453
chrX	17291103	17291554	-1.753073657	B-H1
chr3R	12695005	12695448	-1.753221504	iab-4
chr3L	5603715	5604061	-1.784184103	Eaf6
chr3R	6447868	6448326	-1.809855281	hth
chr3R	12774197	12774737	-1.811020717	Abd-B
chr3L	5884125	5884488	-1.819843841	DNApol-epsilon58
chr3R	2817688	2817988	-1.825223321	Antp
chr2R	10678011	10678429	-1.825251803	kn
chr4	724472	725443	-1.833016621	ey
chr3R	12637409	12637938	-1.854100047	Abd-A
chr3R	2756103	2756553	-1.868966644	Antp
chr3R	2729200	2729631	-1.874411903	Antp
chr2R	15162777	15163289	-1.882906155	rib
chr4	1010061	1010437	-1.913024715	toy
chr2L	1669607	1670095	-1.936337247	chinmo
chr2L	1644318	1644745	-1.947360705	CG31666

* The coordinates are in Dm3, 2006 genome release
